# (Non)linear Interfacial Rheology of Tween, Brij and Span Stabilized Oil–Water Interfaces: Impact of the Molecular Structure of the Surfactant on the Interfacial Layer Stability

**DOI:** 10.1021/acs.langmuir.4c02210

**Published:** 2024-08-10

**Authors:** Kerstin Risse, Stephan Drusch

**Affiliations:** Technische Universität Berlin, Faculty III Process Sciences, Institute of Food Technology and Food Chemistry, Department of Food Technology and Food Material Science, Straße des 17. Juni 135, 10623 Berlin, Germany

## Abstract

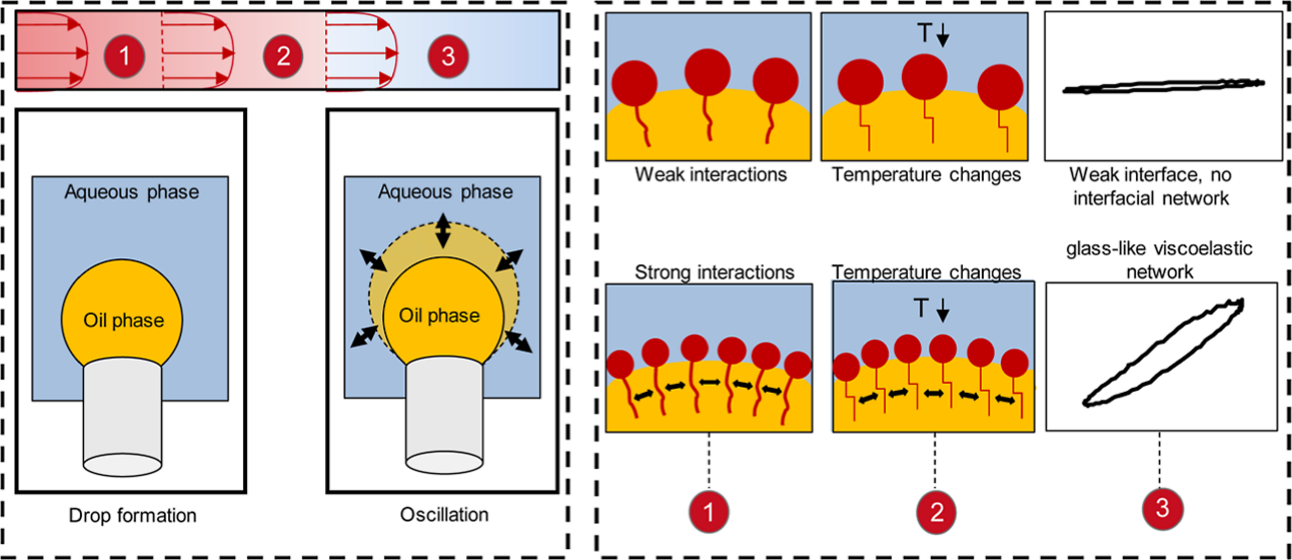

During emulsification and further processing (e.g., pasteurizing), the oil–water interface is mechanically and thermally stressed, which can lead to oil droplet aggregation and coalescence, depending on the interfacial properties. Currently, there is a lack of insights into the impact of the molecular structure (headgroup and FA chain) of low molecular weight emulsifiers (LME) on the resulting interfacial properties. Additionally, the crystallization/melting of the oil/the emulsifier is often neglected within interfacial rheological experiments. Within this study, the stability of interfaces formed by Tween, Span or Brij was determined as a function of their molecular structure, taking crystallization effects of the LME into account. The headgroup was kept constant while varying the FA, or vice versa. The interfacial film properties (viscoelasticity) were investigated at different temperatures using dilatational and interfacial shear rheology. Both the headgroup and the FA chain impacted the interfacial properties. For the same FA composition, a rather small hydrophobic headgroup resulted in a higher packed interface. The interfacial elasticity increased with increased FA chain length (C12 to C18). This seemed to be particularly the case when the emulsifier crystallized on the interface among cooling. In the case of a densely packed interface, network formation due to chain crystallization of the LME’s FA chains occurs during the cooling step. The resulting interface shows predominantly elastic behavior.

## 1 Introduction

Oil–water emulsions are common multiphase systems in the food, cosmetic and pharmaceutical industries and may function as a carrier for bioactive components. Different emulsifiers such as proteins and low-molecular emulsifiers (LME) like phospholipids (such as phosphatidylethanolamine), mono- or diglycerides (glycerol monostearate), different types of Tween and Span are used to stabilize these emulsions.^1^

The emulsification usually takes place within two to three steps. First, the aqueous phase and/or the oil phases are heated to higher temperatures (generally temperature *T* ≥ melting point *T*_m_) to obtain fully melted triglycerides for emulsification and/or to fully dissolve the emulsifier. This step might not be necessary when working with low melting triglycerides. Then, the oil phase is finely dispersed in the aqueous phase by an emulsification step. During this step, the (fully dissolved) emulsifier adsorbs onto the newly created oil–water interface, where it forms an interfacial layer partly covering the droplet. The emulsifier adsorption might be accelerated when working with higher temperatures during emulsification.^2,3^ Finally, the emulsion is cooled down to room temperature (or to lower temperatures), which may lead to a phase transition, i.e. crystallization of the disperse phase and/or the emulsifier. Upon further processing of the emulsion, the interface between oil and water is mechanically stressed, which can lead to aggregation and coalescence of the oil droplets, depending on the interfacial rheological properties. It is generally accepted that a predominantly elastic interfacial layer shows a greater stability toward mechanical stress than a mainly viscous one.^4−7^

Low molecular weight emulsifiers (LME) quickly adsorb on the interface, allowing the production of emulsions with small oil droplet sizes.^8−10^ It is often assumed that LMEs form (ideally) viscous interfacial layers with high fluidity, which are not stable toward stress in the form of droplet deformation.^10−12^ However, for some LMEs, such as Span 65,^13,14^ asphaltenes,^15,16^ oligofructose fatty esters,^17,18^ and saponins,^19^ the formation of viscoelastic interfacial layers has been shown.^13,14,20−24^ Only a limited number of studies in the past decades have tried to link the interfacial behavior of the LME to their molecular structure, but the results are contradictory.^12,25−28^ According to Garti & Sato,^25^ the interactions between the FA chain of the LME and the emulsified triglycerides increase with increasing similarity in FA length and saturation, leading to higher interfacial surfactant concentrations (higher critical micelle concentration, CMC). On the contrary, Hildebrandt et al.^26^ reported that strong interactions with the oil phase can suppress LME adsorption onto the oil–water interface and lower the solubility of LMEs. James-Smith et al.^29^ found a similar behavior and argued that the emulsified triglycerides penetrated the most into the interfacial layer if their *n*-alkane length is similar to the FS chain of the LME due to intermolecular interactions, leading to lower interfacial surfactant concentrations and thus, lower CMCs. This effect is often called chain length compatibility.^27^ Other authors link the amount of LME at the interface to the polarity and the *n*-alkane length of the triglycerides of the emulsified oil only.^12,30^ In the case of emulsified triglycerides with short *n*-alkane length, the area per surfactant molecule decreases, leading to higher CMCs. The intermolecular interactions between the surfactant molecules are stronger than those between surfactant and emulsified triglycerides. Consequently, the oil molecules are squeezed out from the interfacial layer. In the case of triglycerides with long *n*-alkane length, the emulsified triglycerides interact strongly with the hydrophobic surfactant tails. They are incorporated in the adsorption layer, resulting in a higher area per surfactant molecule and lower interfacial surfactant concentrations (lower CMCs).^12^ The same applies to the increasing polarity of the oil phase. More polar oils result in a decrease in the CMC.^12,30−32^

The molecular structure of the LME not only correlates with the interfacial behavior but also affects the crystallization of the dispersed phase during cooling. Depending on the type of LME’s FA and their similarity to the FAs of the disperse phase, the LME may accelerate or decelerate the crystallization of the disperse phase.^33−35^ If the FA chain of the LME has a higher crystallization point than the emulsified triglycerides (*T*_m,LME_ > *T*_m,triglycerides_), the high melting LME may function as a template for heterogeneous crystallization among the cooling step, accelerating the crystallization of the disperse phase.^36−38^ When the FA chain of the LME has a lower melting point than the oil phase, on the other hand, the LME acts as an impurity in the crystallization of the dispersed phase. The result is the formation of less perfect crystals and a loosely packed lattice.^39,40^ Although emulsions usually undergo heating and cooling cycles within industrial processes, temperature alterations are often neglected in interfacial rheological experiments. The multiple phase transitions (melting/crystallization) of the LME/the disperse phase during the production affects the interfacial rheological properties.^40,41^ For instance, interfacial elasticity may increase after a liquid to a solid phase transition due to the molecular ordering or interactions of the LME’s headgroups.^28^ The impact of these temperature alterations on LME interfaces is poorly understood and was therefore evaluated in this study, which is the novelty of this work.

The present study investigates how the molecular structure of the LME affects their interfacial behavior and how the crystallization of the LME upon cooling affects the interfacial rheological properties. LMEs are generally assessed in the linear regime, while nonlinear rheology can provide new insights into the interfacial mechanics. For this reason, we analyze the LMEs in both regimes. Different types of Tween (large, strongly hydrophilic ethoxylated sorbitan headgroup), BrijS20 (large polyethylene glycol headgroup) and Span (small, rather hydrophobic sorbitan headgroup), each with different types of FA chains (see Supporting Information), were used to investigate both, the influence of the headgroup and the role of the hydrophobic FA chain on the resulting interfacial rheological properties. The impact of crystallization of the LME among cooling on the properties of the interface has been analyzed, using medium-chain triglycerides (MCT-oil) as a disperse phase as MCT-oil has a relatively low melting point (>0 °C). First, the long-term adsorption behavior of Tweens, BrijS20 and Spans were analyzed at different temperatures (55, 20 °C) to determine their CMC. The interfacial rheological properties were also investigated at various temperatures (55, 20 °C) using dilatational and interfacial shear rheological measurements.

## 2 Materials and Methods

In the following we give an overview of the used materials and methods. A detailed summary of the different measurements performed (method, short description, surfactant, temperature) can be found in the Supporting Information.

TWEEN 20 (CAS 9005-64-5), TWEEN 60 (CAS 9005-67-8), TWEEN 80 (CAS 005-65-6), SPAN 20 (CAS S8070319), SPAN 60 (CAS S7192421) and SPAN 80 (CAS 4102100219) were purchased from Carl Roth GmbH (Karlsruhe, Germany). Brij S20 (Polyoxyethylene (20) stearyl ether) was purchased from Sigma-Aldrich (St. Louis, MO, USA). The main differences between Tween, BrijS20 and Span are shown in the Supporting Information Medium chain triglyceride oil (MCT-oil) WITARIX MCT 60/40 was kindly provided from IOI Oleo GmbH (Hamburg, Germany). The MCT oil consisted of 0,04% water, 0.1% C 6:0, 56.4% C 8:0, 43.3% C 10:0, 0.1% C 12:0 and 0,0% C14:0. The MCT oil was treated with magnesium silicate (Florisil from Carl Roth GmbH, Karlsruhe, Germany) for 24 h to remove interfacial active substances. The success of the purification step was evaluated using drop tensiometry (constant interfacial tension of 28,16 mN/m ± 2,66 mN/m within the first hour of measurement, *T* = 55 °C).

### 2.1 Sample Preparation

The Tweens and the BrijS20 were diluted at various concentrations in distilled water, while the Spans were diluted in MCT oil. All seven surfactants are nonionic, meaning that the headgroup is noncharged regardless of the pH. Nevertheless, the pH of the aqueous phase was regulated to a pH around 6 for comparability reasons. The surfactant solution (aqueous phase and oil phase) was heated to 55 °C for 30 min prior to the measurement to ensure fully melted FA chains and to exclude crystalline memory effects (molecular arrangement to a previously ordered state during recooling).^42^ The choice of heat treatment was based on DSC measurements (Supporting Information).

### 2.2 Adsorption Behavior of Tweens, BrijS20 and Spans and Determination of the Critical Interfacial Concentration

The adsorption behavior (ability to reduce interfacial tension) of Tweens, BrijS20 and Spans was measured at 55 and 20 °C by drop tensiometry (PAT1M, Sinterface Technologies e.K., Berlin. Germany). The rising drop method was used, where an oil droplet is created at the tip of a curved needle surrounded by the water phase. Crystallization processes at the interface correlate with interface properties such as the storage modulus. Therefore, the rising drop method has been used as the crystallization of emulsified LME differs from that of LME in the bulk phase.^43,44^ The turbidity of the solutions was checked before the start of the experiment to guarantee that the sharpness of the image was not reduced by the liquid surrounding the droplet.^45^

Concentration series of Tweens and BrijS20 were recorded from 0.1, 0.05, 0.01, 0.005, 0.001 to 0.0001% and 1, 0.1, 0.05, 0.01, 0.005 to 0.0001% for the Spans. A 20 mm^3^ droplet of either Span20/Span 60/Span 80 or MCT-oil was formed at the tip of a curved needle with a diameter of 2.989 mm in a cuvette filled with either Tween-water/BrijS20 solution or distilled water (Span).

A high-speed camera was used to take pictures of the given droplet in 40 ms intervals during the entire experiment. A corresponding software (SINTERFACE Profile Analysis Tensiometer PAT1M ver.1.5.0.736) determined the coordinates of the drop profile by image analysis and subpixel edge detection. The interfacial tension was calculated from this information by fitting the Gauss–Laplace Equations (GLE).^46^ The temperature was kept constant at either 55 °C (CMC_55 °C_) or 20 °C (CMC_20 °C_) during the experiment. The interfacial tension was recorded for at least 30 min.

The interfacial tension values were plotted as a function of the concentration, and the CMC was calculated as the intersection of the trend lines.

The area per molecule at the interface was calculated according to^29,30,47,48^ using the Gibbs adsorption isotherm
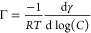
1and
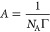
2where Γ is the concentration of surfactant at the interface, *R* is the ideal gas constant, *T* is the temperature, γ is the interfacial tension at the oil/water interface, *C* is the initial concentration of surfactant in the bulk solution, *A* is the area per molecule and *N*_*A*_ is the Avogadro constant. It should be mentioned that there are different models available to calculate the area per molecule and the calculated values will differ from model to model. We worked with the Gibbs adsorption isotherm as this model has proven to be very accurate for nonionic surfactants.^29,30,47−49^

### 2.3 Interfacial Dilatational Rheology of Tweens, Brij and Spans

**Figure 1:**
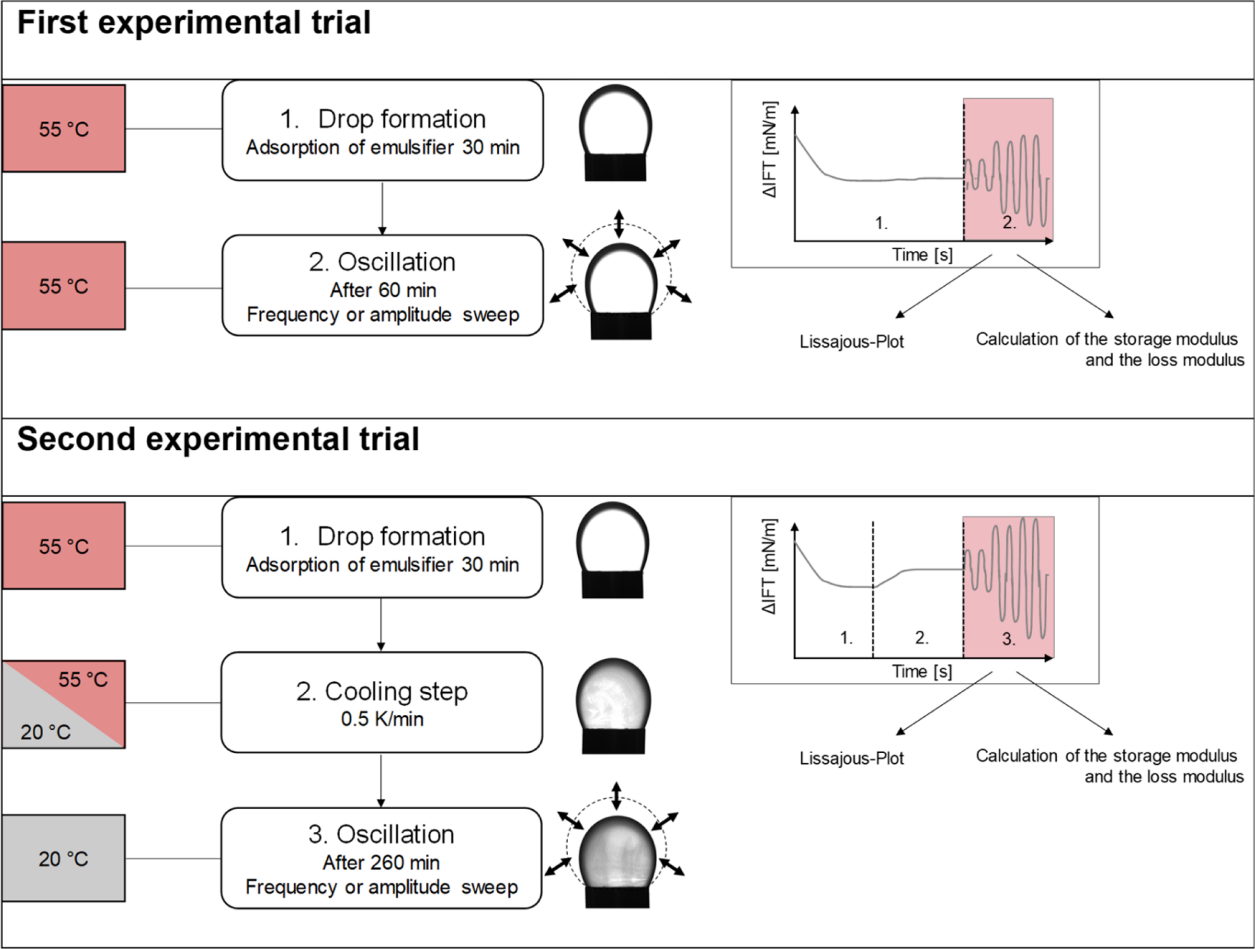
Flowchart of the two experimental trials used.

Dilatational rheology was carried out at the CMC by rising drop tensiometry (PAT1M, Sinterface Technologies, e.K., Berlin, Germany). The interfacial rheological properties were determined at 55 °C (experimental trial 1, fully melted LME) and 20 °C (experimental trial 2, crystalline LME) within two different experimental series (Figure 1). To determine the beginning of crystallization and network formation of Span 60 during cooling, we performed a third experimental trial where we put a sinusoidal oscillation on the created Span 60 drop while cooling down (*T* 55 to 20 °C). Information about the third experimental trial can be found in the Supporting Information. The differences between the first and second experimental trials are shown below. Parameters such as the density of the aqueous and oil phases are needed for the calculation. Once the measurement is started, these parameters cannot be changed. Therefore, only the data from the third section (3. Amplitude Sweep) were evaluated for the second experimental trial, and the density values at 20 °C were used.

The general procedure (Step 1. Drop formation) for the two experimental trials was the same: Tween 20, Tween 60, Tween 80 and the BrijS20 were dissolved in distilled water at their critical micelle concentration (CMC) of ∼0.1 wt % and Span 20, Span 60 and Span 80 were dissolved in MCT-Oil at a concentration of their CMC of ∼0.2 wt %. Then, the solutions (either Tweens, BrijS20 or distilled water (Spans)) were filled in the preheated cuvette, and the temperature was set to 55 °C. A 20 mm^3^ droplet of either Span 20/Span 60/Span 80 or MCT-oil was formed at the tip of a curved needle with a diameter of 2.989 mm in a cuvette filled with either Tween-water/BrijS20 solution or distilled water (Spans). The drop was equilibrated for 1 h (first experimental trial, Figure 1, measurement at 55 °C) or 4 h and 20 min (Second experimental trial, Figure 1, measurement at 20 °C after cooling). Subsequently, a strain amplitude or frequency sweep was carried out. Amplitude sweeps were performed with amplitudes between 1 and 12% at a fixed frequency of 0.01 Hz. Frequency sweeps were performed with frequencies between 0.002 and 0.1 Hz at an amplitude of 1% within a separate measurement. The droplet was subjected to ten cycles at every amplitude or frequency.

The dilatational storage modulus *E*_d_′ and loss modulus *E*_d_″ are calculated from the interfacial data using Fourier transformation as follows
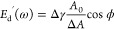
3
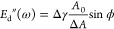
4where ϕ is the phase angle, *A*_0_ is the equilibrium area and γ the interfacial tension.^50^ The phase angle is 0° for an ideal elastic material and 90° for an ideal viscous one. *A* value between 0° and 90° indicates viscoelastic behavior of the interfacial layer.^4^

Lissajous plots were used to describe changes in the microstructure of the interfacial layer during the amplitude sweep as described in the literature.^18,51−53^ For this purpose, the change in interfacial tension (ΔIFT = σ – σ_0,_ with σ_0_ interfacial tension at zero strain) was plotted against the change in the area (Δ*A*/*A*_0_; Δ*A* = *A* – *A*_0,_ with *A*_0_ = area at zero strain = equilibrium area) for different amplitudes. Plots were generated for each deformation from the second oscillation cycle.

### 2.4 Interfacial Shear Rheology of Tween 60, BrijS20 and Span 60

The interfacial shear rheology of the Tweens, BrijS20 and Spans was carried out using the MCR 301 rheometer (Physica Modular Compact Rheometer 301; Anton Paar GmbH; D-Ostfildern) and the RheoCompass Software v1.25. A mini interfacial shear rheology measuring cell (IRS cell mini) with a diameter of *d* = 80 mm and a total height of 28, 2 mm was used. Aliquots of 40 mL of the aqueous phase were filled in the measuring cell, ensuring no air bubbles entered the system. A biconically shaped measuring geometry Bic68-5 with a diameter of 68.183 mm and α of 4983° was used as the measuring body. The bicone was placed at the oil–water interface. Then, 40 mL of the oil phase was added carefully on top of the water phase. The measurement was carried out in oscillation at a constant temperature of 20 °C within three steps: time sweep (film formation), frequency sweep and amplitude sweep. Two different frequencies (0.3 or 0.01 Hz) were tested within the time sweep and amplitude sweep. A high shear rate (high frequency) might interfere with the structure/network formation on the interface (increased nucleation, breakdown of larger aggregates/crystals^54^), while a too-low frequency goes with a low data quality due to the insufficient resolution for the values of torque.^4^ The duration of the time sweep (constant oscillation at 1% and a frequency of either 0.3 or 0.001 Hz) was set at 1 h. The frequency sweep was carried out with frequencies from 0.001 to 1 Hz and a constant amplitude of 0.1% directly after the time sweep. After the frequency sweep, an amplitude sweep was done using a continuous frequency of 0.3 or 0.01 Hz and amplitudes ranging from 0.1 to 100%. The interfacial storage modulus *G*_*i*_′ and loss modulus *G*_*i*_″ were calculated from the deformation default and the time-delayed shear stress response. *G*_*i*_′ and *G*_*i*_″ were plotted toward the deformation (shear rate). The linear viscoelastic (LVE) region was calculated using the 3% deviation of the storage modulus from its original value. The amplitude sweep data were shown in Lissajous plots, which allows the evaluation of the elastic and viscous behavior of the interfacial layer within the LVE and nonlinear viscoelastic range (NL range).

Information about the strain softening/hardening and the strain rate thinning/thickening behavior of the interfacial layer is gained by calculating the nonlinearity parameters *S* (stiffening factor) and *T* (thickening factor). In the literature, *S* (stiffening factor) is defined as follows
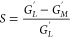
5where *G*_M_′ is the elastic moduli at minimum deformation, and *G*_L_′ is the elastic storage moduli at maximum deformation. The material shows linear viscoelastic behavior when the *S* value is around zero. While strain-softening materials have a negative *S* value, strain-hardening materials have a positive *S* value.^51,55^

*T* (thickening factor) is calculated as follows
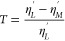
6where η_M_′ is the viscous moduli at minimum deformation, and η_L_′ is the viscous moduli at maximum deformation. The material shows a single harmonic linear viscous response when the *T* value is around zero. A positive *T* value represents intracycle shear thickening, while a negative *T* value indicates intracycle shear thinning.^55^

### 2.5 Statistics

The measurements were carried out at least in triplicate. The mean value was calculated, and the standard deviation was determined.

## 3 Results and Discussion

### 3.1 Adsorption Behavior and Critical Micellar Concentration of Tweens, Brij and Spans

At first, we analyzed the adsorption behavior (ability to reduce interfacial tension) of different Tweens and Spans, by tracking the interfacial tension (IFT) as a function of temperature, concentration and time. Then, the CMC was determined at 20 and 55 °C, and the area per molecule was calculated (Table 1, full curves of the IFT are shown in the Supporting Information).

**Table 1:** Critical Micelle Concentration (CMC), Interfacial Tension (IFT) at the CMC, Concentration of Surfactant at the Interface (Γ) and Area per Molecule of Different Tweens, Spans, and Brij Determined Using Drop Tensiometry^a^

		55°C					20°C				
		IFT at CMC [mN/m]	CMC [wt %]	CMC × 10–^6^ [mol/L]	Γ [μmol/m^2^]	area per molecule [nm^2^]	IFT at CMC [mN/m]	CMC [wt %]	CMC × 10–^6^ [mol/]	Γ [μmol/m^2^]	area per molecule [nm^2^]
ethoxylated sorbitan headgroup	Tween 20 (C12:0)	4.6 ± 0.8	0.01 ± 0.00	8.46 ± 2.01	0.59 ± 0.00	2.82 ± 0.07	5.2 ± 0.1	0.01 ± 0.00	9.22 ± 1.05	0.47 ± 0.00	3.55 ± 0.11
	Tween 60 (C18:0)	6.1 ± 0.2	0.01 ± 0.00	11.54 ± 1.83	0.50 ± 0.00	3.36 ± 0.08	7.6 ± 0.3	0.01 ± 0.00	7.07 ± 1.87	0.34 ± 0.00	4.91 ± 0.55
	Tween 80 (C18:1)	5.7 ± 0.1	0.01 ± 0.01	10.32 ± 0.44	0.59 ± 0.00	2.84 ± 0.00	6.1 ± 0.2	0.01 ± 0.01	8.17 ± 1.56	0.63 ± 0.00	2.85 ± 0.79
sorbitan headgroup	Span 20 (C12:0)	12.1 ± 0.2	0.17 ± 0.11	537.24 ± 3.47	1.38 ± 0.00	1.21 ± 0.05	12.3 ± 0.4	0.19 ± 0.11	600.23 ± 7.50	1.15 ± 0.00	1.45 ± 0.01
	Span 60 (C18:0)	11.5 ± 0.3	0.15 ± 0.09	357.92 ± 1.98	1.38 ± 0.00	1.21 ± 0.08	10.2 ± 0.9	0.25 ± 0.02	591.67 ± 3.48	1.10 ± 0.00	1.52 ± 0.09
	Span 80 (C18:1)	14.4 ± 0.18	0.17 ± 0.05	390.31 ± 0.05	1.32 ± 0.00	1.27 ± 0.03	13.4 ± 0.6	0.21 ± 0.07	494.62 ± 1.70	1.11 ± 0.00	1.51 ± 0.16
glycol headgroup	BrijS 20 (C18:0)	4.8 ± 0.01	0.01 ± 0.02	5.34 ± 1.60	0.20 ± 0.00	8.48 ± 0.56	6.3 ± 0.04	0.01 ± 0.00	5.22 ± 0.33	0.26 ± 0.00	6.56 ± 0.44

a IFT of pure MCT oil, no emulsifier is 28.2 mN/m ± 2.7 mN/m at 55 °C and 25.0 ± 0.1 mN/m at 20 °C

The impact of the FA on the interfacial properties was evaluated by comparing the LME with the same headgroup but different FA tails, so by comparing the Tweens with each other and the Spans with each other. Then, we will identify the effect of the headgroup by comparing Tweens and Spans, which have the same FA tail but vary in headgroup. In this context, we will include BrijS20 within the comparison to consider the effect of the hydrophobicity of the headgroup.

At 55 °C the IFT of the Tweens was around 4.6 to 6.1 mN/m, while at 20 °C the IFT was around 5.2 to 7.6 mN/m (Table 1) (as a comparison IFT of pure MCT oil, no emulsifier was around 28.2 ± 2.7 mN/m at 55 °C and 25.0 mN/m ± 0.1 at 20 °C). It was assumed that the CMC would decrease with increasing temperature as some of the existing hydrogen bonds between water molecules and LME’s headgroup would rupture due to thermal fluctuation, favoring micellization.^56^ Nevertheless, the CMC of the Tweens was around 10 μmol/L at both temperatures, indicating that the temperature had little to no impact on the hydration of the hydrophilic oxyethylene groups, and, therefore the adsorption behavior remained unchanged. At both temperatures, the Tween with the shortest FA, Tween 20, led to the lowest IFT, which has been described in the literature before.^57^ The ability of a LME to reduce interfacial tension increases with increasing similarity to the oil phase.^25^ It was assumed that the CMC would decrease with increasing LME’s FA chain length due to the decreasing hydrophilicity of the molecule.^49,56,58^ Nevertheless, regardless of the FA, all three Tween had a CMC of around 10 μmol/L.

With a range from 10.2 to 13.4 mN/m (T 20 °C), the IFT of Span also varied in a relatively narrow range. Interestingly, the CMC of Span 60 (591.67 μmol/L) was slightly higher than those of Span 80 (494.62 μmol/L). A possible explanation could be the long, nonbended (saturated) FA chain of Span 60, which favored LME–LME interactions. In the case of the unsaturated FA chain of Span 80, the bend in the molecule acted as a placeholder for the emulsified triglycerides within the interfacial layer, lowering the CMC and increasing the area per molecule as expected.^59^

Surprisingly, the IFT and CMC of the Tweens are much lower, and the area per molecule is larger than those of the Spans, regardless of the FA. A more important factor with respect to IFT and CMC is the difference in the structure of the hydrophilic headgroup between Span and Tween. Unlike Tweens, Spans have a small, rather hydrophobic headgroup with no ethylene groups, which allowed a closer packaging at the interface (lower area per molecule, higher CMC) but hindered strong interaction with the aqueous phase as a result of the lower amount of hydrophilic groups within the LME headgroup (higher IFT). A similar trend was recently shown by Bergfreund et al.^30^ for anionic (SDS), cationic (DTAB), and nonionic (Brij 35) surfactants. The adsorption of the oil-soluble Spans onto the oil–water interface might have additionally been limited due to interactions within the oil phase.^26^ The lower packing density of the Tweens is probably the reason why no impact of the FA on IFT or CMC was detected for this group of LMEs. The large headgroup of the Tweens hindered the Tween’s FA from getting close enough in contact with each other to interact. Accordingly, the FA only impacts the final interfacial rheological properties when the area the headgroup occupies on the interface is not too large. The effect of the headgroup was further confirmed through an investigation of BrijS20 (similar CMC and final IFT), which a headgroup with higher hydrophobicity and comparable size to Tween 60.

### 3.2 Interfacial Dilatational Rheology of Tweens, Brij and Spans

Dilatational rheology was performed to investigate the stress behavior of the LME layer at a curved interface with particular emphasis on the temperature-induced crystallization of the LME. In this context, amplitude sweeps with amplitudes in a range of 1–12% were carried out. The results are presented in Lissajous plots. In a Lissajous plot, a perfect circle represents ideal viscous behavior, while a straight line represents ideal elastic behavior (elastic shear stress). For viscoelastic materials, the straight line widens with increasing viscous portions and becomes ellipsoidal. The slope of the ellipse/the straight line correlates with the storage modulus. The steeper the slope, the higher the storage modulus.

In the following, we will only discuss the interfacial rheological properties at an amplitude of 7.8% (biggest differences in interfacial structures). Higher deformations could not be applied since, at amplitudes above 7.8%, the deformations in the neck region of the droplet deviated significantly from those in the apex region, and the droplet shape could no longer be fitted by the Young–Laplace equation with sufficient accuracy^60^ (standard error from Young–Laplace equation larger than ±0.05). Amplitudes below 7.8% can be found in the Supporting Information.

**Figure 2:**
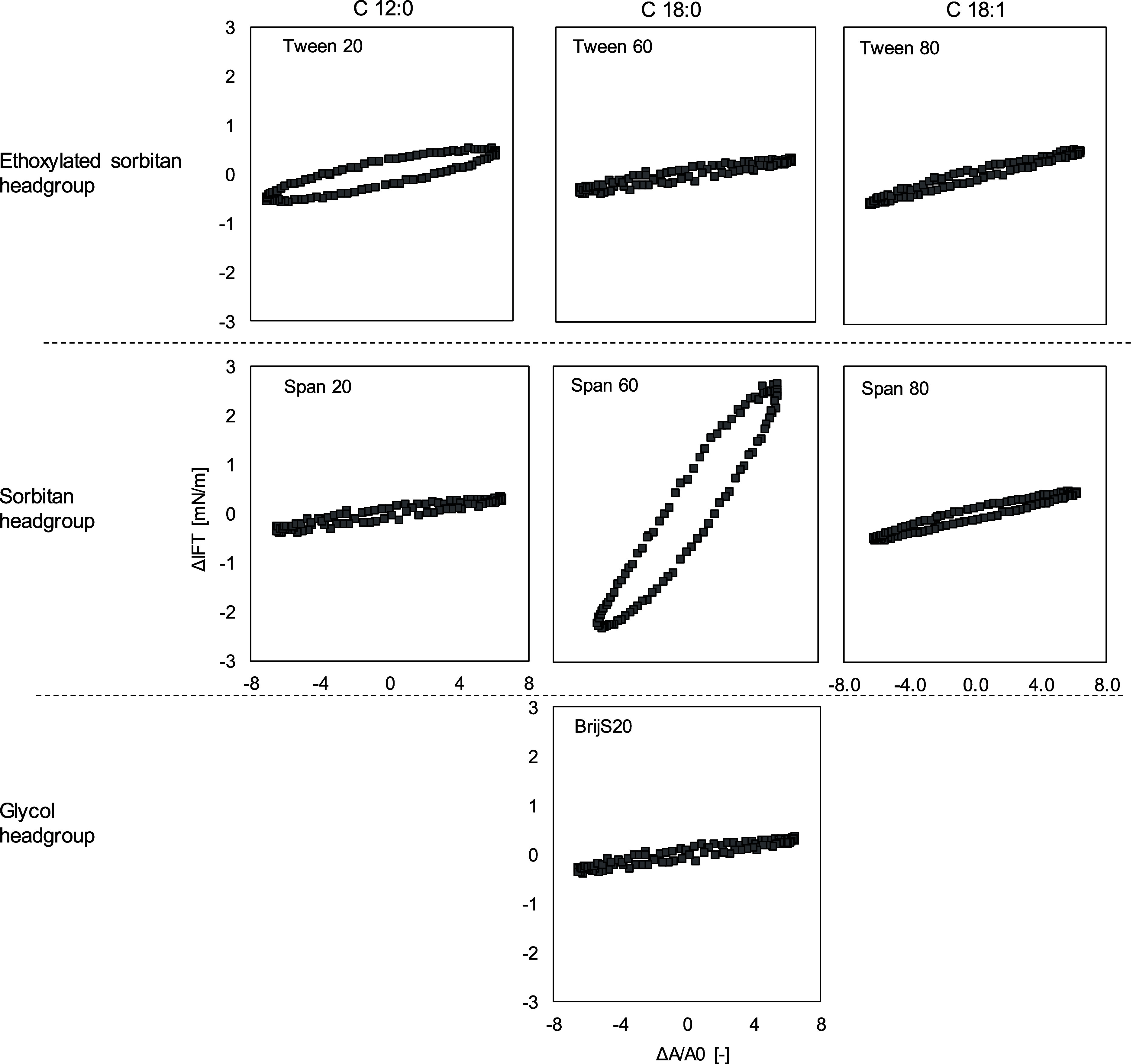
Lissajous plots of interfacial tension versus deformation obtained during amplitude sweeps (1–12%) of aged oil–water interfacial stabilized by Tweens, Spans or BrijS20 at *T* = 20 °C and a frequency of 0.01 Hz. Lissajous plots plotted as Δσ (Δ*A*/*A*_0_) plots to assess the elasticity of the interface at an amplitude of 7.8%. Interfacial rheological properties were determined at the LME’s CMC: Tween 20:9.22 μmol/L. Tween 60:7.07 μmol/L. Tween 80:8.17 μmol/L. BrijS20:5.22 μmol/L, Span 20:600.23 μmol/L, Span 60:591.67 μmol/L, Span 80:494.62 μmol/L, respectively.

Figure 2 shows Lissajous plots of Tweens, Spans or BrijS20 stabilized oil–water interfaces at *T* = 20 °C. Tweens (Tween 20, Tween 60, Tween 80) have a constant dilatational storage modulus (*E*_d_′) [mN/m] and loss modulus (*E*_d_″) [mN/m] over the chosen amplitudes (Supporting Information). We observe *E*_d_′ > *E*_d_″ for all three Tweens, indicating predominantly elastic behavior, which is shown in the Lissajous plots by the ellipsoidal curves (Figure 2). The values for *E*_d_′ and *E*_d_″ are relatively low (*E*_d_′ Tween 20–8 mN/m, *E*_d_′ Tween 60–4,7 mN/m, *E*_d_′ Tween 80–8 mN/m), and the corresponding Lissajous plots are flat, indicating that these LME did not form networks on the interface, regardless of the FA chain. Therefore, the predominantly elastic response might be caused by the exchange of Tweens between the bulk and the interface upon deformation. This aspect will be further discussed at the end of this chapter (frequency sweeps, Figure 4).

A widening in the curves was observed in the Lissajous plots of Tween 20 (C 12 FA) compared to Tween 60 or Tween 80 (C 18 FA), implying a less elastic interface, possibly due to weaker LME–LME interactions, which slow down the reformation process after deformation.^61^ Finally, when comparing Tween 60 (C18:0) and Tween 80 (C18:1), the storage modulus and the slope of the Lissajous plots of Tween 80 were slightly higher compared to Tween 60. The unsaturated hydrophobic group of Tween 80 is more flexible than the saturated C18 FA residue of Tween 60. During the droplet expansion and compression, the FA chains of Tween 80 got cross-linked and entangled due to the bend in the FA residue.^57^ Additionally, the C18:1 FA chains might have interacted via π-interactions, leading to a higher storage modulus and, thus, a stiffer interface.^26,62^

As for the Tweens, the values for *E*_d_′ and *E*_d_″ for Span 20 and Span 80 are low (*E*_d_′ Span 20–3.5 mN/m, *E*_d_′ Span 80–7 mN/m), and the curve in the Lissajous plots linear but flat, suggesting that no network formation took place (Figure 2). Conversely, the *E*_d_′ and *E*_d_″ of Span 60 are significantly higher (*E*_d_′ around 40 mN/m), and the slope of the Lissajous plots is larger than those of the other Spans, implying strong LME–LME interactions on the interface. The stronger microstructure of Span 60 could be related to the difference in FA and the accompanying difference in *T*_m_ compared to the other Spans. Measurements with DSC (see Supporting Information) suggest that Span 20 (C12:0 FA) and Span 80 (C18:1 FA) are still liquid at an experimental temperature of 20 °C, resulting in an easily stretchable interface during the dilatation of the drop^a^.

In contrast, Span 60 is already crystalline at this temperature (crystallization point 40–45 °C). It has been reported that LMEs can form a two-dimensional network on the interface upon cooling due to associative interactions between the LME’s FA chains, leading to chain crystallization.^35,63^ This network formation leads to an increased *E*_d_′ and accordingly, a greater slope in the Lissajous plots.^4,64^ The periodic expansion and compression of the droplet size result in the partial breakdown of the crystalline network, causing a widening of the Lissajous plots due to internal friction.^4^ During the compression, wrinkles appeared in the interfacial layer (Supporting Information), indicating very limited desorption of Span 60 back into the bulk during oscillation due to the high interface binding energy.^65^

While network formation was detected for Span 60, no network formation was seen in dilatation for Tween 60, although these two LMEs have the same FA chain. A more important factor with respect to the strength of the interface is, therefore, not the FA chain alone but the difference in the structure of the hydrophilic headgroup between Span and Tween. According to measurements with the DSC (Supporting Information), crystallization of both LMEs may occur during the cooling step, which was also seen optically (Supporting Information). The small headgroup allowed the FA chains of Span 60 to interact with each other strongly during the cooling step, while the distance between the FA chains was too large in the case of Tween 60 for strong interactions to occur, and thus, no network formation took place. This effect did not depend on the hydrophobicity of the headgroups, as shown by the comparison with BrijS20 (*E*_d_′_BrijS20_ ∼ 2,6 mN/m vs *E*_d_′_Tween60_ ∼ 4,7 mN/m). It is assumed that Tween 60 (and BrijS20) molecules are presented as single molecules on the interface.

**Figure 3:**
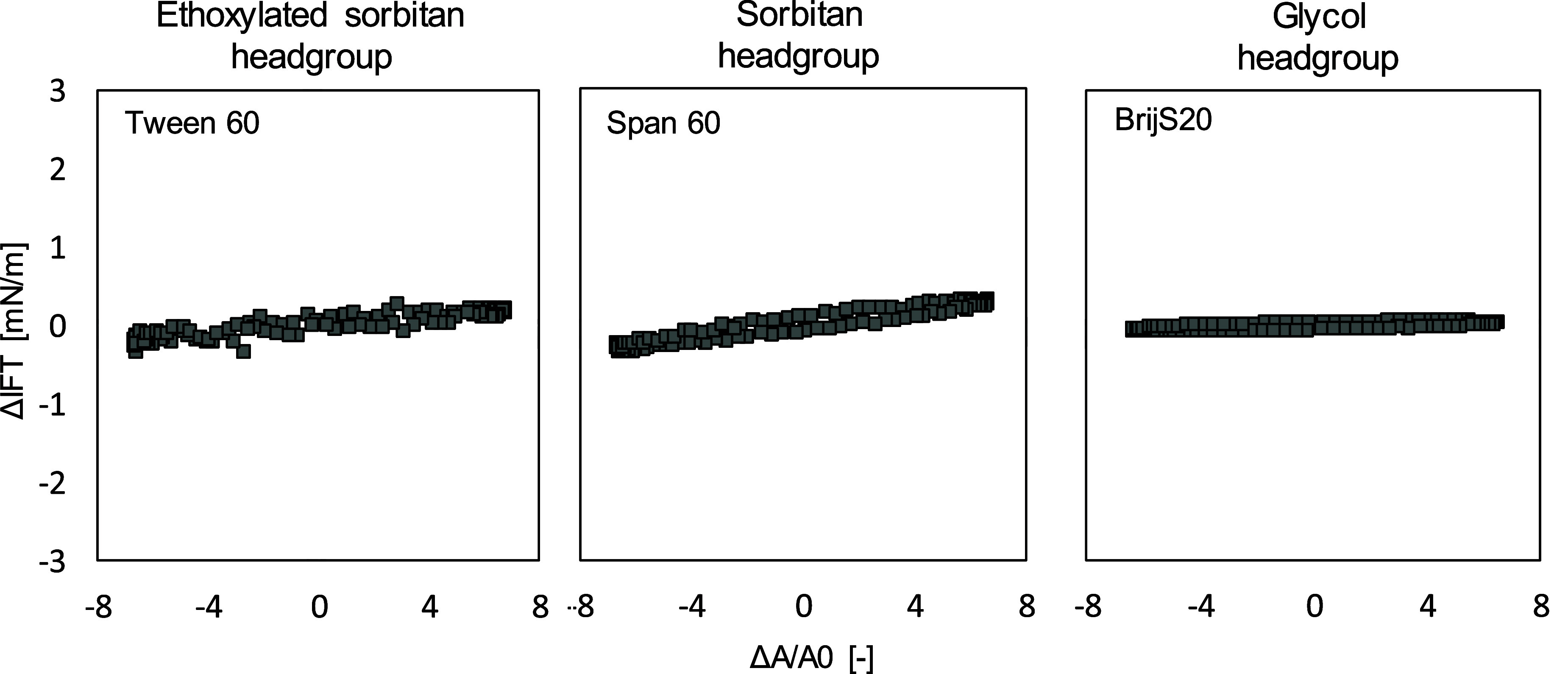
Lissajous plots of interfacial tension versus deformation obtained during amplitude sweeps (1–12%) of aged oil–water interfacial stabilized by Tween 60, Span 60 or BrijS20 at *T* = 55 °C at a frequency of 0.01 Hz. Lissajous plots plotted as Δσ(Δ*A*/*A*_0_) plots to assess the elasticity of the interface at an amplitude of 7.8%. Interfacial rheological properties were determined at the LME’s CMC: Tween 60:7.07 μmol. BrijS20:5.22 μmol/L, Span 60:591.67 μmol/L, respectively.

To investigate the impact of crystallization of the LME on interfacial rheological properties, amplitude sweeps were additionally performed at 55 °C (fully melted LME) for Tween 60, Span 60 and BrijS20 stabilized oil/water interfaces. In the following, only the results of these three LMEs will be discussed since crystallization effects (and a possible network formation) were only observed for LMEs with a C18:0 FA. In this context, we will compare Tween 60, Span 60 and BrijS20 to identify the impact of the headgroup on the final interfacial rheological properties. The results of the amplitude sweep at 55 °C are shown in Figure 3 again for an amplitude of 7.8%.

At a temperature of 55 °C, we observed an *E*_d_′ and *E*_d_″ around one mN/m (storage and loss moduli curves see Supporting Information) and flat but linear Lissajous plots for all three LMEs. This deformation behavior might be caused by the high fluidity and mobility of the LME molecules. Other researchers have shown that the temperature influences the stability of interfacial layers.^1,57^ At high temperatures, the interfacial dilatational viscosity of the oil phase is lower, resulting in a lower resistance of the interface against continuous expansion and, therefore, a lower strength of the interface, as observed within our study.

The low slopes of the Lissajous plots (slopes close to zero) indicate the formation of easily stretchable interfaces, i.e. no network formation took place, not even in the case of Span 60, implying that the strong microstructure of Span 60 at 20 °C builds up among cooling. To determine the beginning of crystallization and network formation of Span 60 during cooling, we performed a third experimental trial where we put a sinusoidal oscillation on the created Span 60 drop while cooling down (T55 to 20 °C, Supporting Information). At 23–26 °C, we observed a sudden increase in *E*_d_′, implying that the phase transition i.e. network formation occurred at this temperature range.

**Figure 4:**
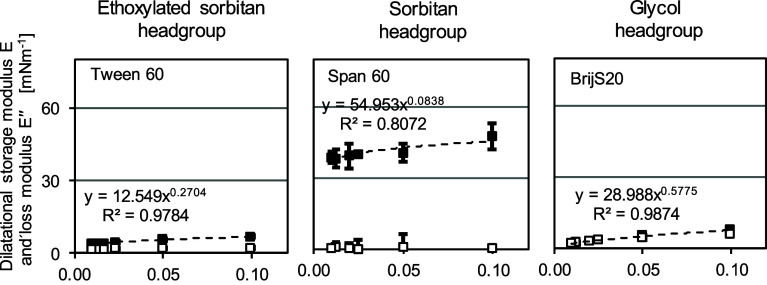
Storage modulus *E*_d_′ and *E*_d_″ [mN m^–1^] of Tween 60 (left), BrijS20 (middle) and Span 60 over frequency, with a fixed amplitude of 1% using the drop tensiometer in emergent drop mode at *T* = 20 °C. Interfacial rheological properties were determined at the LME’s CMC: Tween 60:7.07 μmol/L. BrijS20:5.22 μmol/L, Span 60:591.67 μmol/L, respectively.

To identify whether exchange processes with the bulk phase contribute to the elastic response in the Lissajous plots, frequency sweeps in the range of 0.002 and 0.1 Hz were performed at a temperature of 20 °C and a fixed amplitude of 1% (Figure 4).

In the frequency sweeps of Tween 60, BrijS20 and Span 60, we observe that the *E*_d_′ is larger than the *E*_d_″, which implies predominantly elastic behavior (as seen in the Lissajous plots).

The *E*_d_′ and *E*_d_″ of Tween 60 and BrijS20 are relatively low and frequency-dependent, as indicated by the high value of the exponent of the trend line of *E*_d_′ (*n*-value) (Figure 4). According to the Lucassen-Van den Tempel model,^66^ a value of *n* ∼ 0.5 indicates that the elasticity of the interface is mainly dominated by the exchange of LME between the bulk and the interface. For BrijS20 interfaces, we observe a much stronger frequency dependency (*n*-value ∼0.6) compared to Tween 60 (*n*-value ∼0.3), suggesting the formation of a more viscous interfacial structure by the BrijS20, possibly due to less in-plane LME interactions. The headgroup of Tween 60 was the largest of all three LMEs. It is hypothesized that the ethylene oxide groups of the Tween 60 headgroup formed a sublayer on the interface, which interlinks partly during oscillation. These interactions, however, were not large strong enough to cause permanent network formation, leading to relatively low values for the storage modulus. The headgroup of BrijS20 was less branched and less hydrophobic. For this reason, the headgroup of this LME did not interlink nor interact significantly; it was thermodynamically more favorable to desorb/adsorb during oscillation, as indicated by the high *n*-value.^66^ This assumption is confirmed by the high phase angle of BrijS20 (highest phase angle over amplitude compared to Tween 60 and Span 60, Supporting Information).

The *E*_d_′ and *E*_d_″ of Span 60 remained constant over the range of frequencies, and the resulting *n*-value is 0.08 (Figure 4), which means that the deformation behavior of Span 60 is not influenced by the frequency. The low *n*-value of Span 60, hence, implies that the contribution of diffusional exchange of Span 60 between the bulk and the interface to the response is negligible, which was previously shown for gel-like and soft glassy materials on interfaces stabilized by proteins.^18,67,68^

To confirm that no network formation on the interface occurred in the case of Tween 60 and BrijS20 and to analyze the disordered solid interfacial structures of Span 60, interfacial shear rheology was performed and will be discussed in the following.

### 3.3 Interfacial Shear Rheology of Tween 60, BrijS20 and Span 60

**Figure 5:**
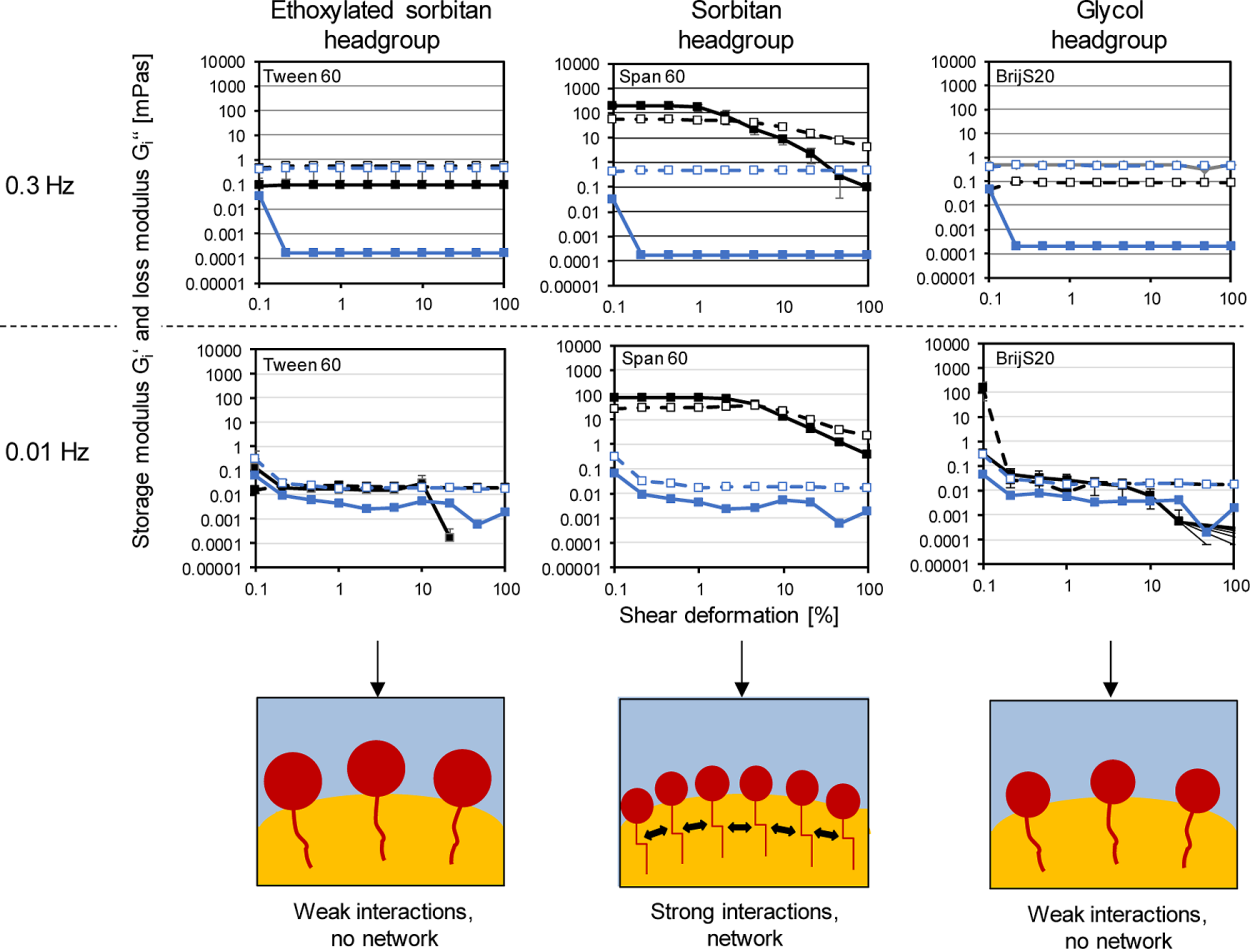
Interfacial storage modulus *G*_*i*_′ and Lost modulus *G*_i_″ [mPa] of Tween 60 (left), Span 60 (middle) and BrijS20 (right) over amplitude, with a fixed frequency of 0.3 Hz (1, upper raw) and 0.01 Hz (2, lower raw). The blue lines represent the *G*_*i*_′ and *G*_i_″ of the reference measurement (oil–water interface without any LME). Interfacial rheological properties were determined at the LME’s CMC: Tween 60:7.07 μmol/L. BrijS20:5.22 μmol/L, Span 60:591.67 μmol/L, respectively.

**Figure 6:**
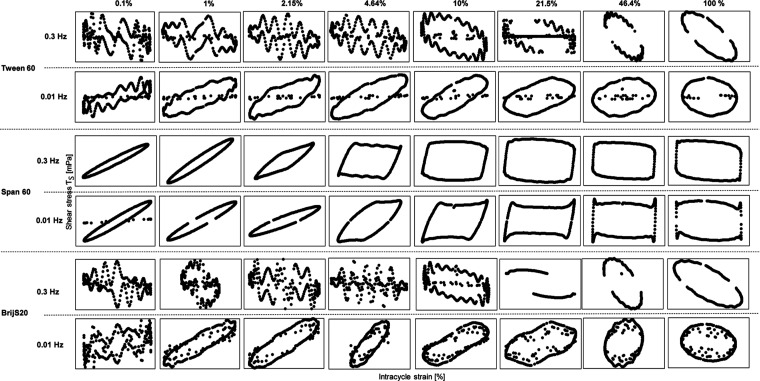
Stressing of the aged LME interfacial layer on the Water MCT oil interface at *T* = 20 °C by interfacial shear rheology using the rheometer. Lissajous plots plotted as strain plots for amplitudes of 0.1, 1, 2.15, 4.64, 10, 21.5, 46.4 and 100% to analyze the elasticity of the Tween 60, Span 60 and BrijS20 layers at a frequency of 0.3 Hz (upper raw) and 0.01 Hz (bottom raw). Interfacial rheological properties were determined at the CMC (591.67 μmol/L).

Figure 5 shows the Interfacial storage modulus *G*_*i*_′ and lost modulus Gi” of Tween 60, BrijS20 and Span 60 over amplitude (0.1–100%) at 0.3 and 0.001 Hz frequencies, and Figure 6 shows the corresponding Lissajous plots at different amplitudes (0.1, 1, 2.12,4.64,10, 21.5, 46.4, 100%).

At a frequency of 0.3 Hz, we observe a G_i_″ above *G*_*i*_′ for Tween 60 and BrijS20 (Figure 5), indicating viscous deformation behavior as shown in the Lissajous plots by the large area within the ellipse (Figure 6). The fluctuations in the curves within the Lissajous plots imply that the contribution of the subphase drags to the measured torque signal was relatively large compared to the torque stress value generated by the interface, leading to the noise in the signal.^69^ Hence, it is assumed that no network formation took place. However, all LMEs showed viscoelastic behavior in dilatational rheology (Figure 2).

On the one hand, in dilatational rheology, exchange processes with the bulk phase contributed to the elastic response in the Lissajous plots of Tween 60 and BrijS20, as shown by the frequency sweeps. On the other hand, the compression of the oil droplet within dilatational rheology might have favored weak interactions between the LMEs by pushing the LME molecules closely together, allowing headgroup chains to entangle or interlink. These interactions were not large enough to cause network formation.

The interfacial layer formation was measured again via interfacial shear rheology for the Tween 60, BrijS20 and Span 60-stabilized interfaces at a lower frequency (0.01 Hz) to analyze whether the network formation was interfered with (or even inhibited) by the oscillation of the bicone. However, the output was the same. The LME with large headgroups did not form a network on the interface and behaved predominantly viscous in shear.

Network formation was only observed for the Span 60 stabilized interface (for both frequencies). For Span 60, we observe a constant *G*_*i*_′ and G_i_″ until a critical strain (3% decrease of *G*_*i*_′ from max strain, linearity limit) of 1% (Figure 5). This is shown in the Lissajous plots by a predominantly elastic response (small ellipsoidal curves with rather large slope) (Figure 6). The formation of disordered solid structures at the interface was confirmed via a frequency sweep (Supporting Information). The Span 60 stabilized interface showed a deformation behavior, which is not influenced by the frequency (*n* value of 0.2), which was already seen in dilatational rheology. At strains above the critical strain, *G*_*i*_′ starts to decline in dependence on the frequency (steeper slope for 0.3 Hz). We observe *G*_*i*_′ > *G*_*i*_″ in the LVE regime, suggesting solid-like behavior possibly due to the strong LME–LME interactions. After the critical strain, the Lissajous plots become highly nonlinear and the area between the curves increases, meaning that the interface becomes more viscous. Surprisingly, Span 60 first shows a weak strain overshoot, which is classified as weak type III nonlinear behavior (*G*_*i*_′ decreasing, *G*_*i*_″ increasing),^70^ given by the balance between the breakdown and formation of network junctions, where new junctions result from cluster collisions.^52^ When increasing the strain even further, after an amplitude of 4.64% (0.3 Hz) and 9.99% (0.01 Hz), we observe strain thinning (*G*_*i*_′, *G*_*i*_″ decreasing) as a result of the breakdown of the clusters. In the Lissajous plots, the slope of the ellipse decreases, and the curves become rhomboidal, indicating a predominantly elastic response, until the stress equals the yield stress, after which the structure temporarily breaks down, and the interface begins to flow.^21,52^ For the low frequency, the shape of the curves gets pointy at the edges of the rhombus compared to the Lissajous plots at the same amplitude but at a higher frequency of 0.3 Hz. This could be due to the relaxation of the interface, as the frequency was so low it gave the interface time to recover from the deformation. A frequency of 0.3 Hz was, thus, already enough to interfere with the structure formation during the time sweep (first step of the measurement, previous to amplitude sweep; surfactant adsorption and (re)arrangement processes on the newly created interface).

**Figure 7:**
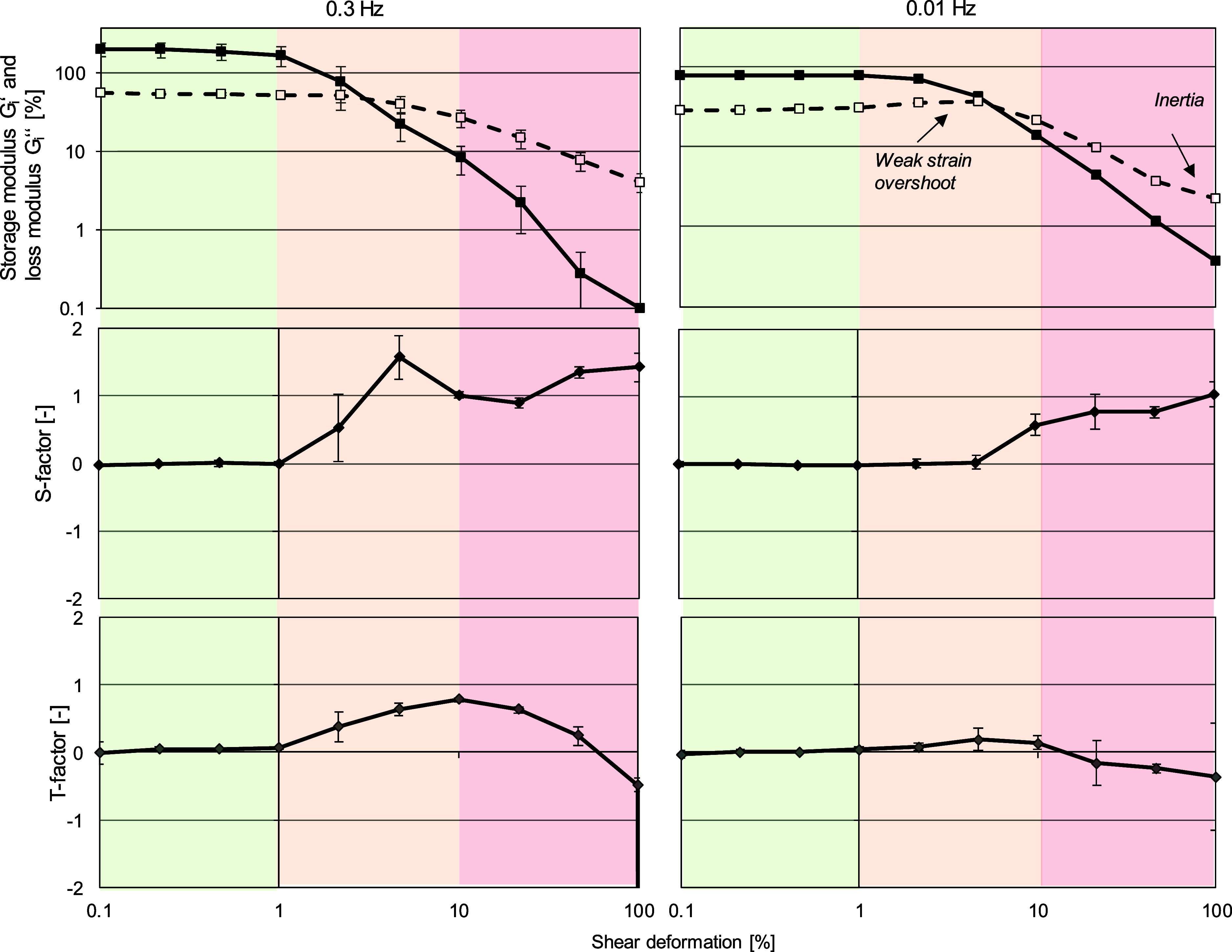
*S*-factor and *T*-factor determined from strain sweeps of Span 60 at a frequency of 0.3 Hz (right) and 0.01 Hz (left). Measurements were performed at 20 °C. Interfacial rheological properties were determined at the CMC (591.67 μmol/L).

To quantitatively study the Lissajous plots, we have determined the nonlinearity parameters S (stiffening factor) and T (thickening factor), as shown in Figure 7. Only the frequency sweep of Span 60 will be discussed below, as Tween 60 and BrijS20 showed predominantly viscous behavior in shear.

For amplitudes lower than 1%, the S-factor and T-factor are both around 0, which indicates linear viscoelastic behavior. This behavior was already seen in Figure 5, where we detected a linearity limit of 1%. After reaching the linearity limit (>1%), we observe a positive *S*-factor that increases with increasing amplitude, indicating local strain hardening. The apparent strain hardening is visible in the Lissajous plot on the curve for the largest strain amplitude as a slight upturn at high strain.^71^ The strain hardening might be given by the balance between the breakdown and formation of network junctions, where new junctions result from cluster collisions,^52^ as discussed above. For large deformations, after the interfacial structure yields, the elastic moduli at minimum deformation *G*_M_′ has a value near zero. The elastic moduli at maximum deformation *G*_L_′ will determine the *S*-factor, leading to a positive S-factor equal to strain hardening.

On the other hand, the *T*-factor increases slightly until an amplitude of 10%. This behavior can be interpreted as follows: among low amplitudes (>1%), we observe intracycle shear thickening until the linearity limit is reached, and the interfacial structure partly breaks down. After that, the material shows intracycle shear thinning.

After an amplitude of 10%, the *S*-factor suddenly increases again while the T-factor decreases. Taking Figures 5 and 7 into account, we assume that the increasing S-factor/decreasing *T*-factor are an effect of inertia.

## 4 Conclusion

We have studied the interfacial behavior (adsorption behavior in the sense of ability to reduce interfacial tension, interfacial rheological properties) of seven low molecular weight emulsifiers (LME), namely Tween 20, Tween 60, Tween 80, BrijS20, Span 20, Span 60 and Span 80, as a function of their molecular structure and the temperature. Either the headgroups or the fatty acid chain (FA) were kept constant to investigate the influence of both the headgroup and the FA. Before/after emulsification, the emulsion is usually heated/cooled down to cause the melting/crystallization of the oil phase. The impact of these temperature alterations on interfaces is poorly understood and was evaluated in this work. The interfacial rheological properties were investigated at different temperatures (55, 20 °C) using dilatational and interfacial shear rheological measurements.

Comparing the critical micelle concentration (CMC) of Span, Tween and Brij with similar FA chains, it becomes obvious that a relatively small hydrophobic headgroup facilitates a more densely packed interface than a rather large and hydrophilic headgroup of the LME. Contrary to other findings,^12,25−27^ in this study, the CMC was not affected significantly by the LME’s FA, suggesting that the polarity and the *n*-alkane length of the triglycerides of the emulsified oil are more crucial for the CMC than the LME’s FA.^12,30^

At high temperatures, the interfacial layers were very unstable in dilatational rheology, regardless of the headgroup or the FA. The LME’s molecular motion is faster at high temperatures, decreasing in-plane interactions on the interface between LME molecules and, consequently, increasing the likelihood of LME desorption when mechanical stress is applied to the system. In the same way, the interfacial viscosity is lowered, allowing a fast incorporation/removal of the LME. Subsequently, the elasticity of the interfaces increased with decreasing temperature, which is in agreement with other studies.^1,57^ During cooling, physical changes occur on the interface. Consequently, the formation of a crystalline LME network on the interface due to chain crystallization was possible during the cooling step (LMEs with C18:0 FA) if the amount of surfactant on the interface was large enough (Span 60). A highly packed interface was only reached for LMEs with small headgroups. The effect does not depend on the hydrophobicity of the headgroup, as shown by a comparison of Tween, Span and Brij with similar FA (C18:0). The crystallization and network formation of Span 60 on the interface increased the dilatational storage modulus and, consequently, the stability of the interfacial layer toward mechanical stress.

In general, the elastic modulus increased when replacing C12:0 with C18:0 in the LME, possibly due to stronger LME–LME interactions on the interface. For LMEs with an unsaturated FA (C18:1), intermolecular entanglement may stabilize the interfacial layer.^57^ In the case of a large headgroup, where crystallization of C18:0 did not lead to a network formation, an increase in the elastic modulus was observed due to this entanglement. In the case of a small headgroup, entanglement does not stabilize the interface as efficiently as chain crystallization and, thus, a decrease in the elastic modulus was observed.

Interfacial shear rheology outside the LVE confirmed the higher stability in dependence on chain length, saturation and size of the headgroup.

A crystalline LME network on the interface might be of interest when crystallization processes within the emulsified oil can occur. A tightly packed crystalline LME network may accelerate the crystallization of the disperse phase as the crystalline LME’s FA might work as a template for heterogeneous crystallization if the LME has a higher crystallization temperature than the emulsified oil.^33,44,72^ This aspect should receive attention in future studies.

## Supporting Information Available

Regarding the molecular structure of Tween 60, BrijS20 and Span 60; CMC determination (IFT curves at the different concentrations), dilatational storage and loss modulus over amplitude (dilatational rheology), phase angle as well as corresponding Lissajous plots at different amplitudes for all seven surfactants; pictures of a Tween 60 drop during cooling (crystallization of surfactant); pictures of Tween 60, BrijS20 and Span 60 during oscillation; development of dilatational storage and loss modulus during cooling of Span 60; Storage modulus *G*_*i*_″ and *G*_*i*_″ of Span 60 over frequency (IRS); DSC measurements of all seven surfactants (heating/cooling curves). (PDF)

## Abbreviations

CMC: critical micelle concentration
DSC: differential scanning calorimetry
FA: fatty acid
LME: low-molecular emulsifiers
LVE: linear viscoelastic
NL: nonlinear viscoelastic

## List of symbols

*T*_m_: melting point
Γ: concentration of surfactant at the interface
*R*: ideal gas constant
*T*: temperature
γ: interfacial tension at the oil/water interface
*C*: initial concentration of surfactant in the bulk solution
*A*: area per molecule
*N*_*A*_: Avogadro constant
*E*_d_′: dilatational storage modulus
*E*_d_″: dilatational loss modulus
*A*_0_: equilibrium area
ϕ: phase angle
*S*: stiffening factor
*T*: thickening factor
*G*_*i*_′: interfacial storage modulus
*G*_*i*_″: interfacial loss modulus
*G*_M_′: elastic moduli at minimum deformation
*G*_L_′: elastic moduli at maximum deformation
η_M_′: viscous moduli at minimum deformation
η_L_′: viscous moduli at maximum deformation

## Footnotes

a Please note the DSC measurements were carried out with the raw materials, i.e., the LME was measured in bulk without first dissolving them in water/oil. It is known that the crystallization of emulsified LME starts at lower temperatures than the crystallization in bulk. Although we do not know the precise crystallization temperature for Span 20 and Span 80 on the interface, we conclude that they must have been in a liquid state at the chosen experimental temperature of 20 °C, as the crystallization of Span 20 and Span 80 in bulk occurs at temperatures below 20 °C, meaning that the crystallization of Span 20 and Span 80 on the interface would need to start at even lower temperatures (*T*_experiment_ (20 °C) > *T*_crystallization of LME in bulk_ (below 20 °C)> *T*_expected crystallization point of the LME on the interface_).
