# Supplementary material for: (Non)linear Interfacial Rheology of Tween, Brij and Span Stabilized Oil–Water Interfaces: Impact of the Molecular Structure of the Surfactant on the Interfacial Layer Stability
Source: Langmuir. 2024 Aug 10;40(34):18283–96. doi: 10.1021/acs.langmuir.4c02210 (PMC11363120; doi:10.1021/acs.langmuir.4c02210)
Supplement: Supplementary file 1 — la4c02210_si_001.pdf [file la4c02210_si_001.pdf]

Supplemented material

(Non)linear interfacial rheology of Tween, Brij and Span stabilised oil-water interfaces: Impact of the molecular structure of the surfactant on the interfacial layer stability

Kerstin Risse[1], Stephan Drusch[1]

[1] Technische Universität Berlin, Faculty III Process Sciences, Institute of Food Technology and Food Chemistry, Department of Food Technology and Food Material Science, Straße des 17. Juni 135, 10623 Berlin, Germany
Corresponding Author: Kerstin Risse
E-mail: k.risse@tu-berlin.de Tel.: +49 (0)30 314-70609

Summary of the different measurements performed (method, short description, surfactant, temperature)

Method	Short description	Surfactant	Temperature
**CMC determination via drop tensiometryy**	Tracking of the IFT as a function of time and concentration and the values were used to calculate the CMC as well as Γ and Area per molecule [nm^2]	Tween 20<br>Tween 60<br>Tween 80<br>Span 20<br>Span 60<br>Span 80<br>BrijS20	55°C, 20°C
**Dilatational rheology (amplitude sweeps) via drop tensiometry**	The interfacial rheological properties of the surfactant interface were determined within an amplitude sweep. In addition, the IFT value was determined 30 minutes after drop formation (step 1, time sweep, before oscillation) and is shown in Table 1.	Tween 20<br>Tween 60<br>Tween 80<br>Span 20<br>Span 60<br>Span 80<br>BrijS20	20°C
**Dilatational rheology (amplitude sweeps) via drop tensiometry After a time sweep, no cooling step**	The interfacial rheological properties of the surfactant interface were within an amplitude sweep. In addition, the IFT value was determined 30 minutes after drop formation (step 1, time sweep, before oscillation) and is shown in Table 1.	Tween 60<br>Span 60<br>BrijS20	55°C
**Dilatational rheology (frequency sweeps) via drop tensiometry After a cooling step and a time sweep**	Frequency sweeps were performed at 20°C after a time sweep and a cooling sweep (separate measurement). The general procedure was analogue to the previous dilatational experiments, but instead of an amplitude sweep, a frequency sweep was carried out.	Tween 60<br>Span 60<br>BrijS20	20°C
**Dilatational rheology (oscillation with fixed amplitude and fixed frequency during the time sweep) DURING the cooling step**	The interfacial rheological properties of the surfactant interface were determined DURING the cooling step by applying a sinusoidal oscillation on the drop during the whole measurement (cooling down step).	Span 60	Temperature sweep from 55°C to 20°C DUIRNG the measurement
**Interfacial shear rheology (frequency and amplitude sweep)**	Interfacial rheological properties were additionally determined via IRS. Therefore, first a time sweep, then a frequency sweep	Tween 60<br>Span 60<br>BrijS20	20°C

	and then an amplitude sweep were carried out.

Supporting information about the molecular structure of the surfactants to illustrate their main differences

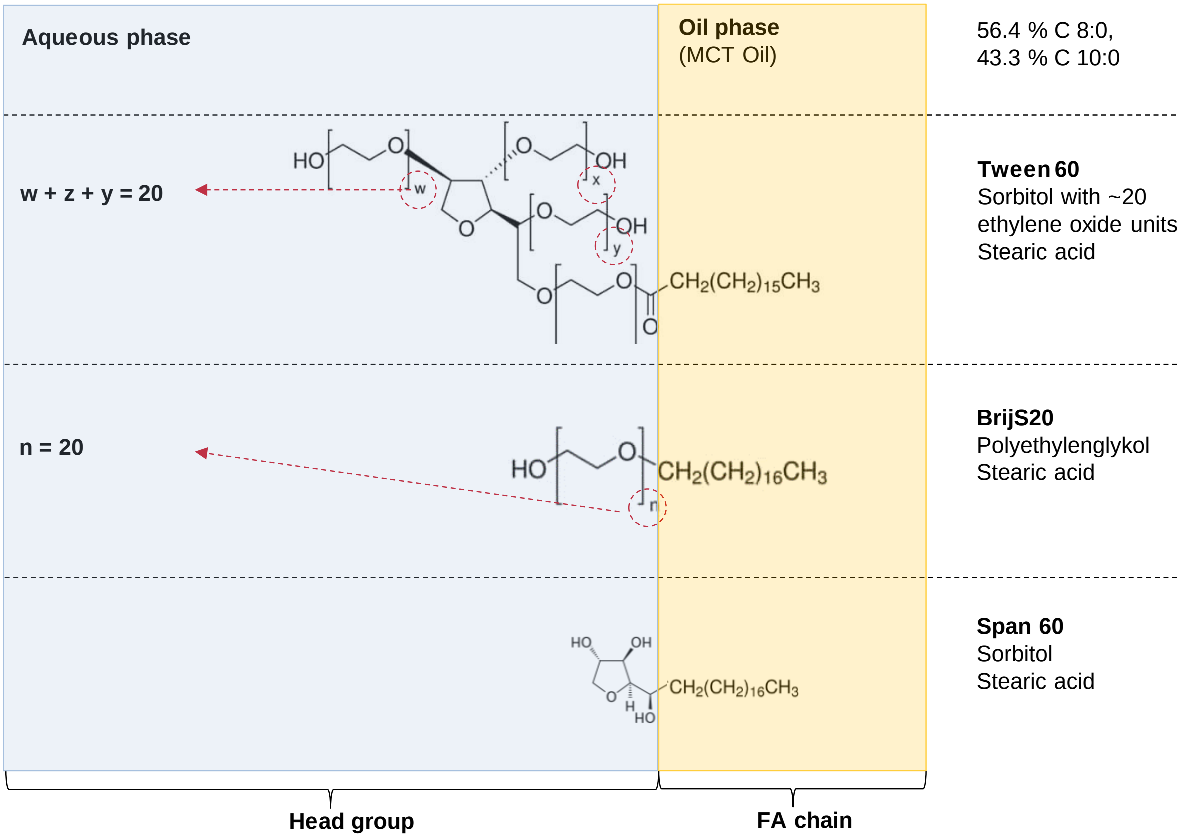

Molecular structure of Tween 60, BrijS20 and Span 60. Blue: hydrophilic part (headgroup) of the surfactant. Yellow: hydrophobic part (fatty acid chain).

CMC determination (tracking of the IFT as a function of LME concentration and time)

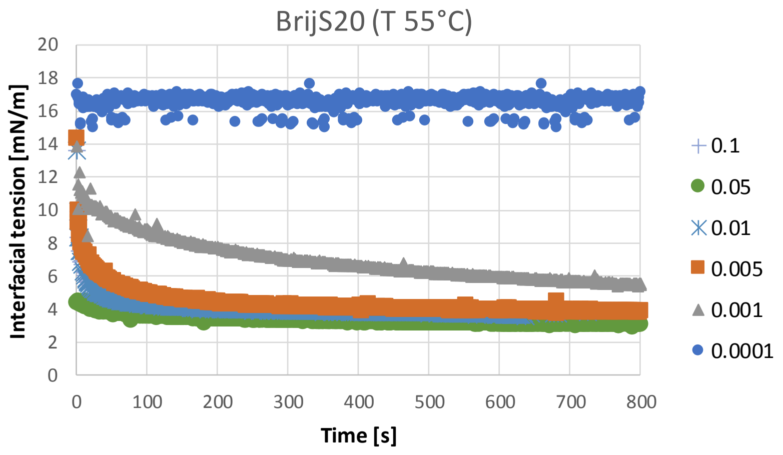

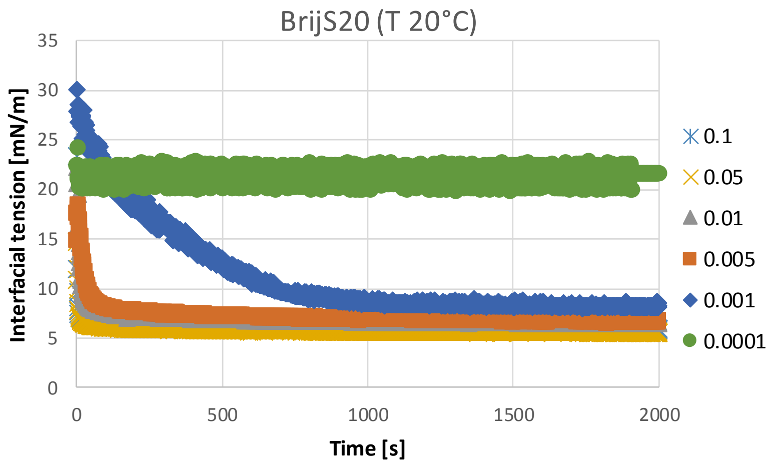

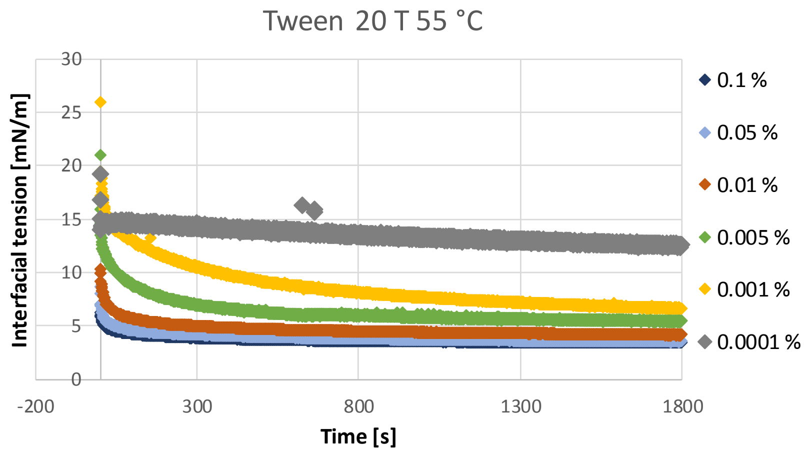

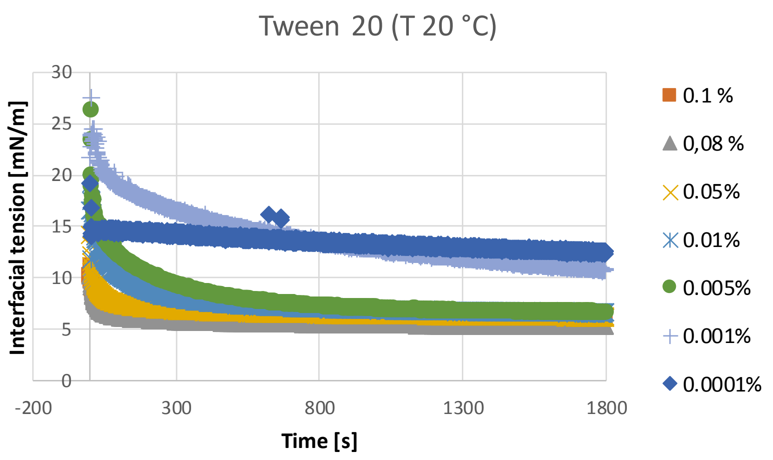
Tween 20 (T 20 °C)
Interfacial tension [mN/m]
Time [s]
30
25
20
15
10
5
0
-200
300
800
1300
1800
0.1 %
0,08 %
0.05%
0.01%
0.005%
0.001%
0.0001%

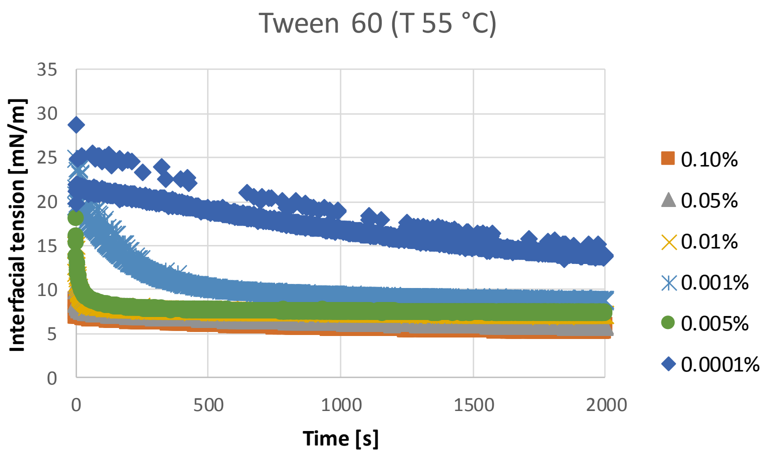
Tween 60 (T 55 °C)
Interfacial tension [mN/m]
Time [s]
35
30
25
20
15
10
5
0
0
500
1000
1500
2000
0.10%
0.05%
0.01%
0.001%
0.005%
0.0001%

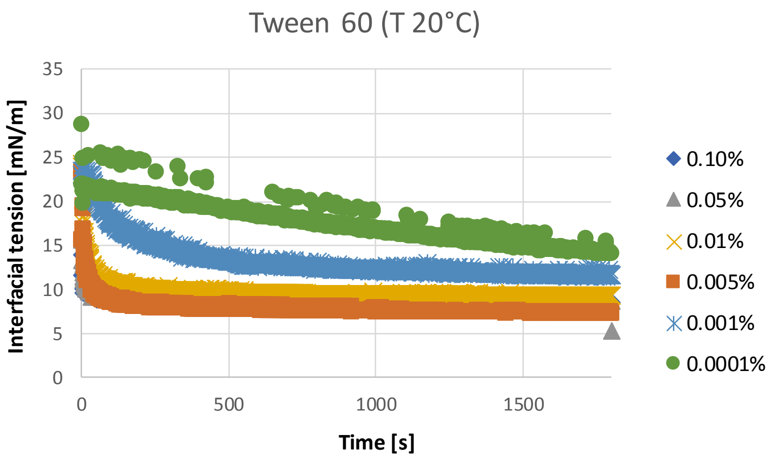
Tween 60 (T 20°C)
Interfacial tension [mN/m]
Time [s]
35
30
25
20
15
10
5
0
0
500
1000
1500
0.10%
0.05%
0.01%
0.005%
0.001%
0.0001%

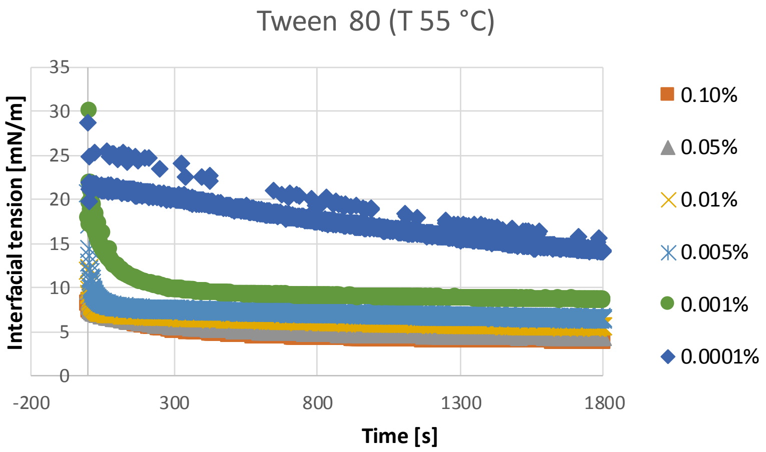
Tween 80 (T 55 °C)
Interfacial tension [mN/m]
Time [s]
0.10%
0.05%
0.01%
0.005%
0.001%
0.0001%

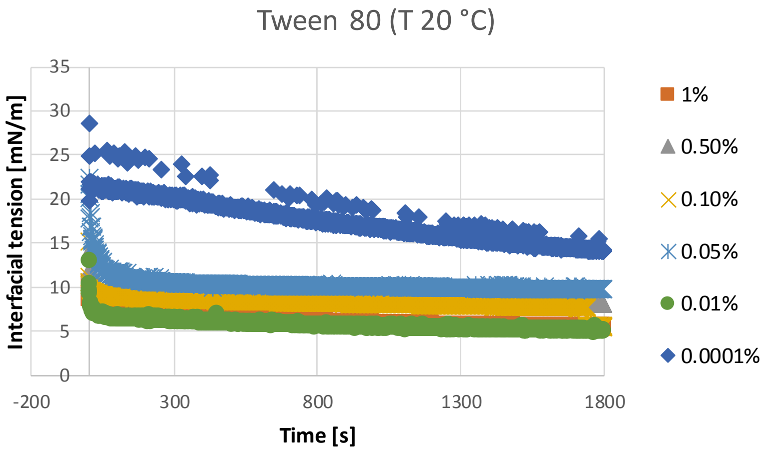
Tween 80 (T 20 °C)
Interfacial tension [mN/m]
Time [s]
1%
0.50%
0.10%
0.05%
0.01%
0.0001%

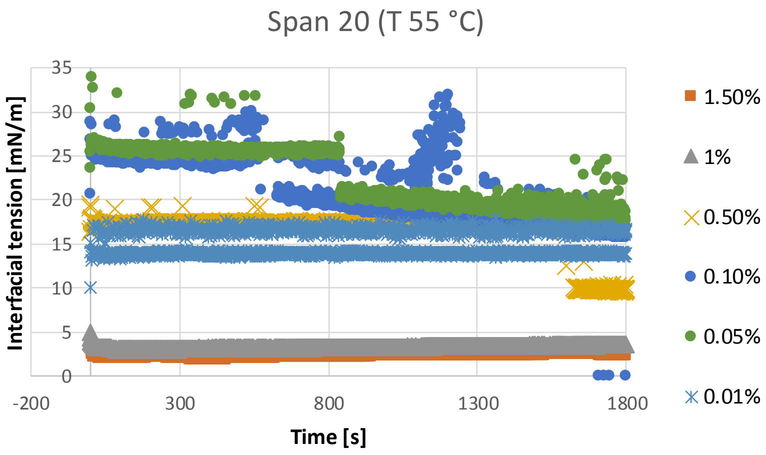
Span 20 (T 55 °C)
Interfacial tension [mN/m]
Time [s]
1.50%
1%
0.50%
0.10%
0.05%
0.01%

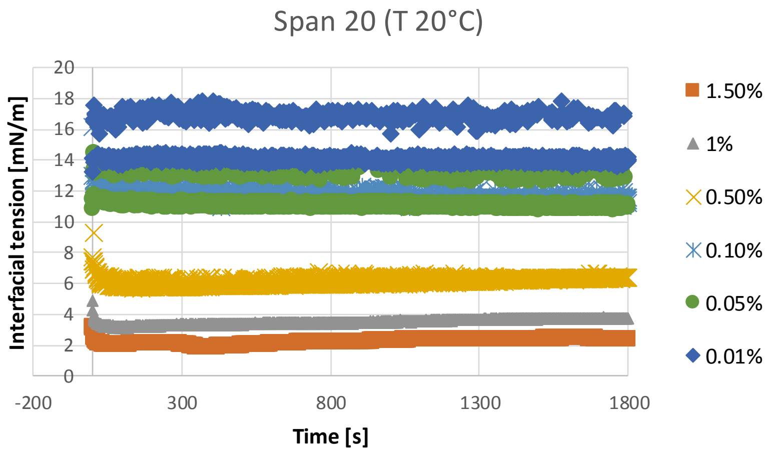
Span 20 (T 20°C)
Interfacial tension [mN/m]
Time [s]
1.50%
1%
0.50%
0.10%
0.05%
0.01%

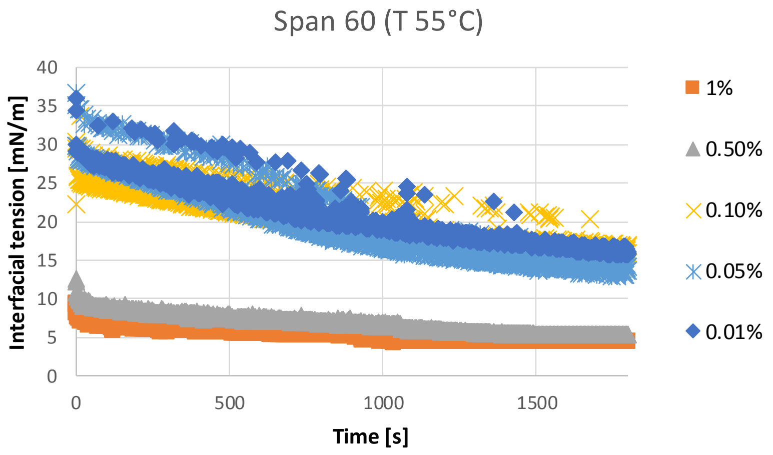
Span 60 (T 55°C)
Interfacial tension [mN/m]
Time [s]
1%
0.50%
0.10%
0.05%
0.01%

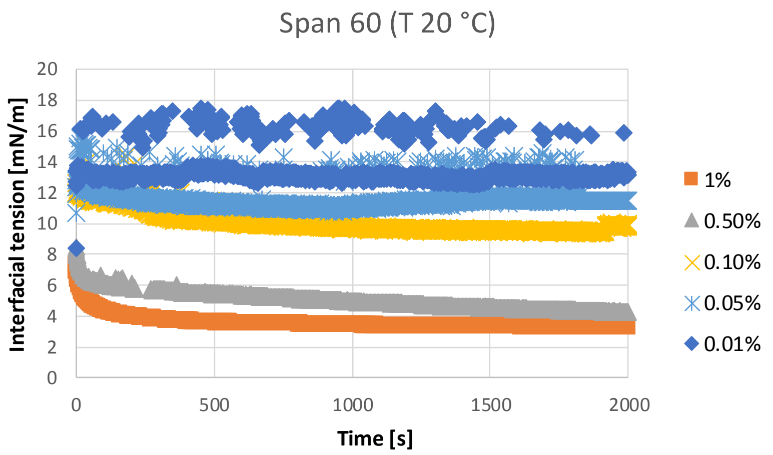
Span 60 (T 20 °C)
Interfacial tension [mN/m]
Time [s]
1%
0.50%
0.10%
0.05%
0.01%

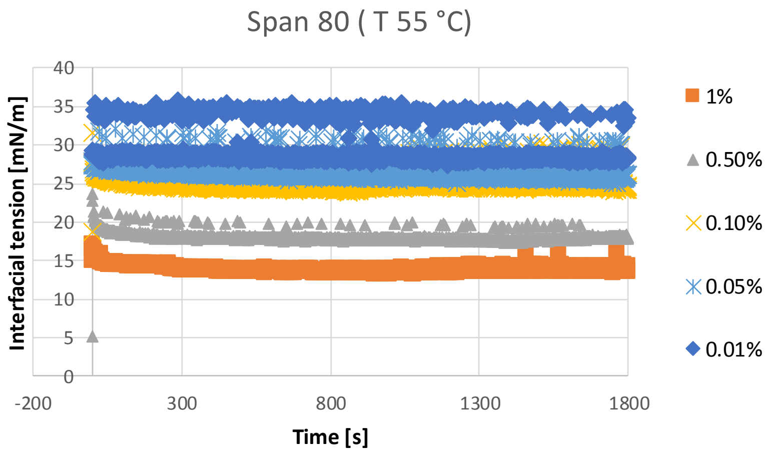

Span 80 (T 55 °C)
Interfacial tension [mN/m]
40
35
30
25
20
15
10
5
0
-200
300
800
1300
1800
Time [s]
1%
0.50%
0.10%
0.05%
0.01%

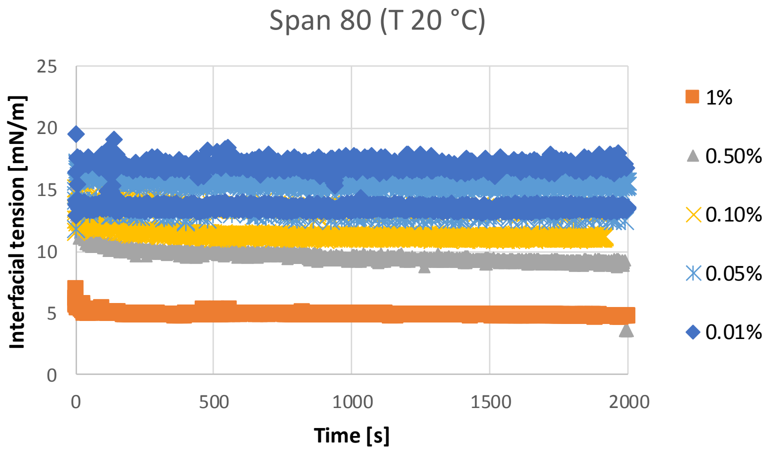

Span 80 (T 20 °C)
Interfacial tension [mN/m]
25
20
15
10
5
0
0
500
1000
1500
2000
Time [s]
1%
0.50%
0.10%
0.05%
0.01%

Dilatational rheology

Storage and loss moduli obtained during amplitude sweeps (0.8-7.8%)

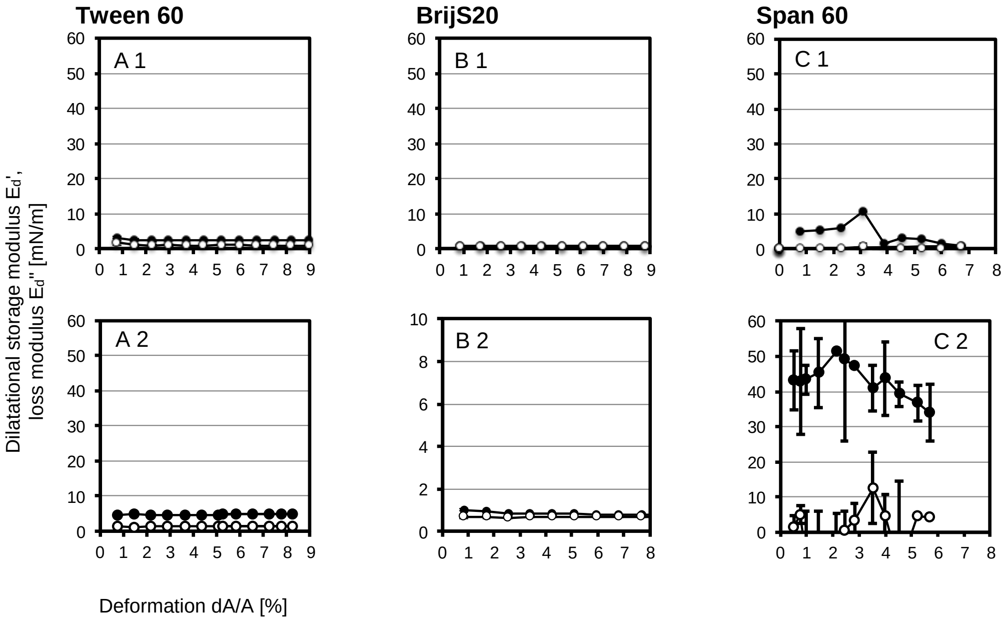

Storage modulus E' and E'' [mNm^{-1}] of Tween 60 (A, left) and BrijS20 (B, middle) and Span 60 (C, right) over amplitude, with a fixed frequency of 0.01 Hz using the drop tensiometer in rising drop mode at T = 55°C (1, upper raw) and T = 20°C (2, lower raw).

Phase angle [°] over amplitude for different Tweens, Spans and BrijS20

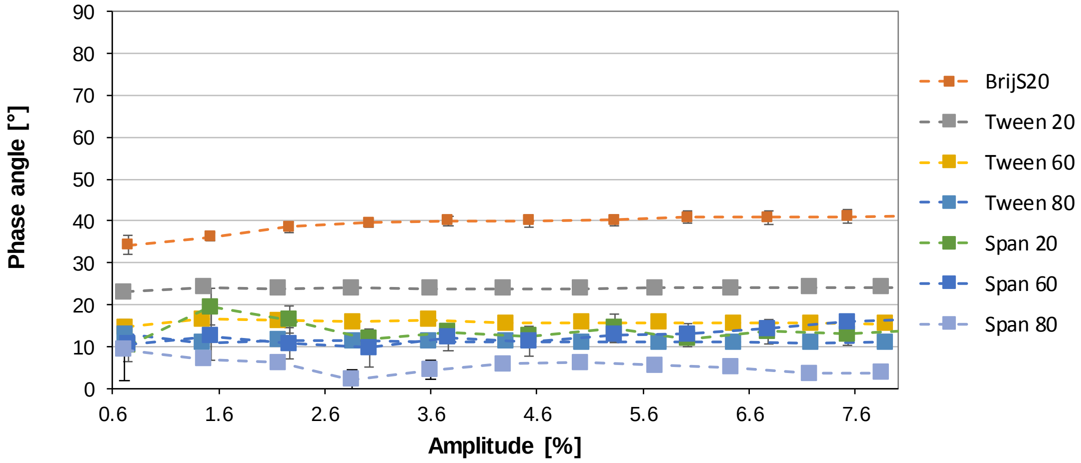

Phase angle [°] over amplitude for BrijS20 (orange), Tween 20 (grey), Tween 60 (yellow), Tween 80 (light blue), Span 20(green), Span 60 (dark blue), Span 80 (blue-grey)

Lissajous plots obtained during amplitude sweeps (0.8-7.8%)

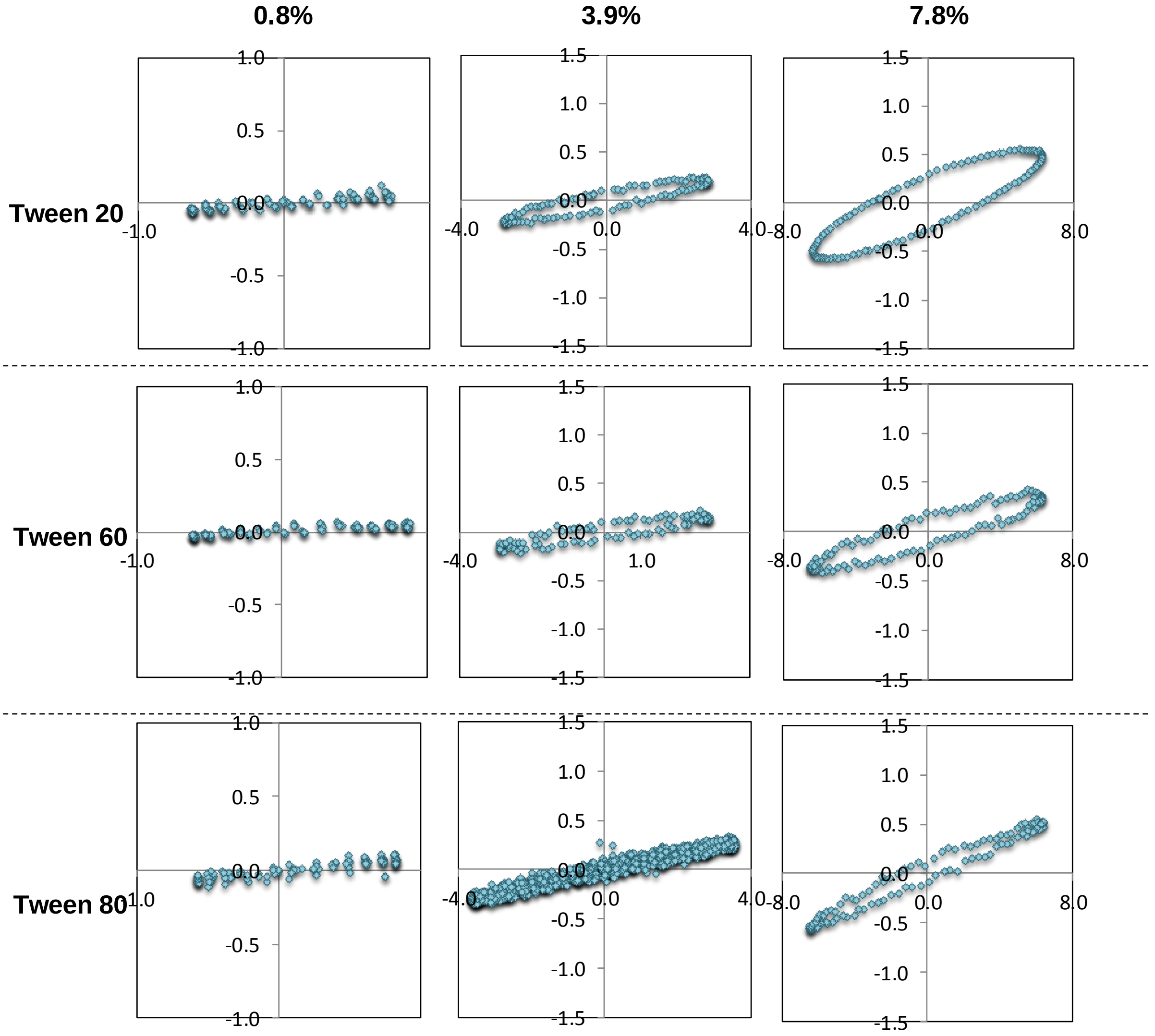

Lissajous plots of interfacial tension versus deformation obtained during amplitude sweeps (0.8-7.8%) of oil/water interfaces stabilised by different Tweens at a frequency of 0.01 Hz.

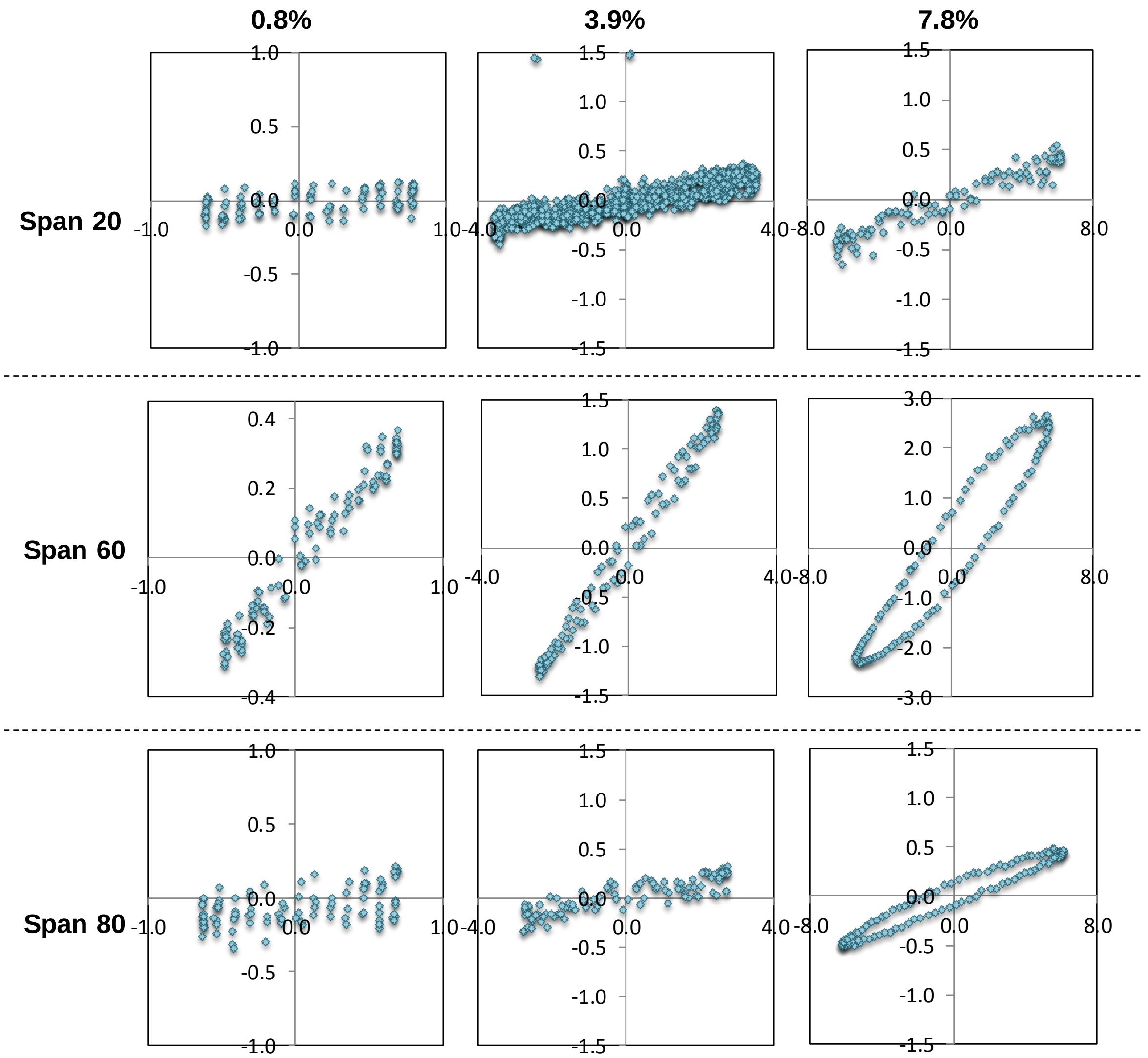

Lissajous plots of interfacial tension versus deformation obtained during amplitude sweeps (0.8-7.8%) of oil/water interfaces stabilised by different Spans at a frequency of 0.01 Hz.

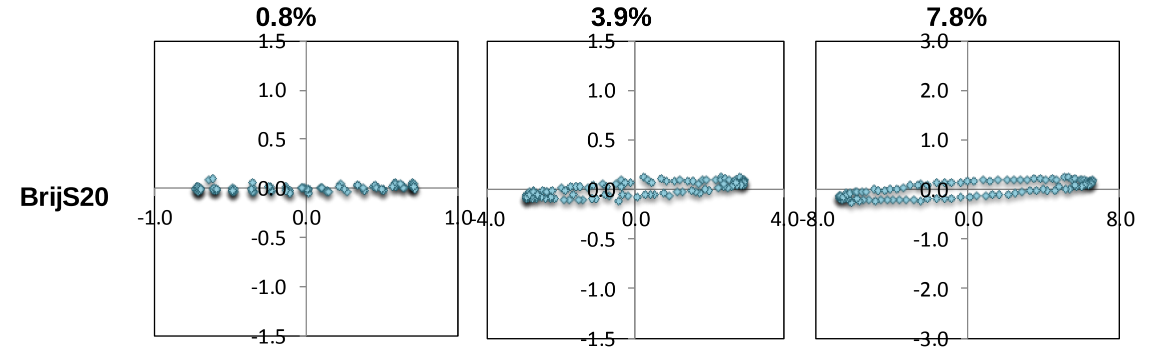

Lissajous plots of interfacial tension versus deformation obtained during amplitude sweeps (0.8-7.8%) of oil/water interfaces stabilised by BrijS20 at a frequency of 0.01 Hz.

Changes of the oil droplet among cooling

Tween 60 drop during cooling (55°C to 20°C)

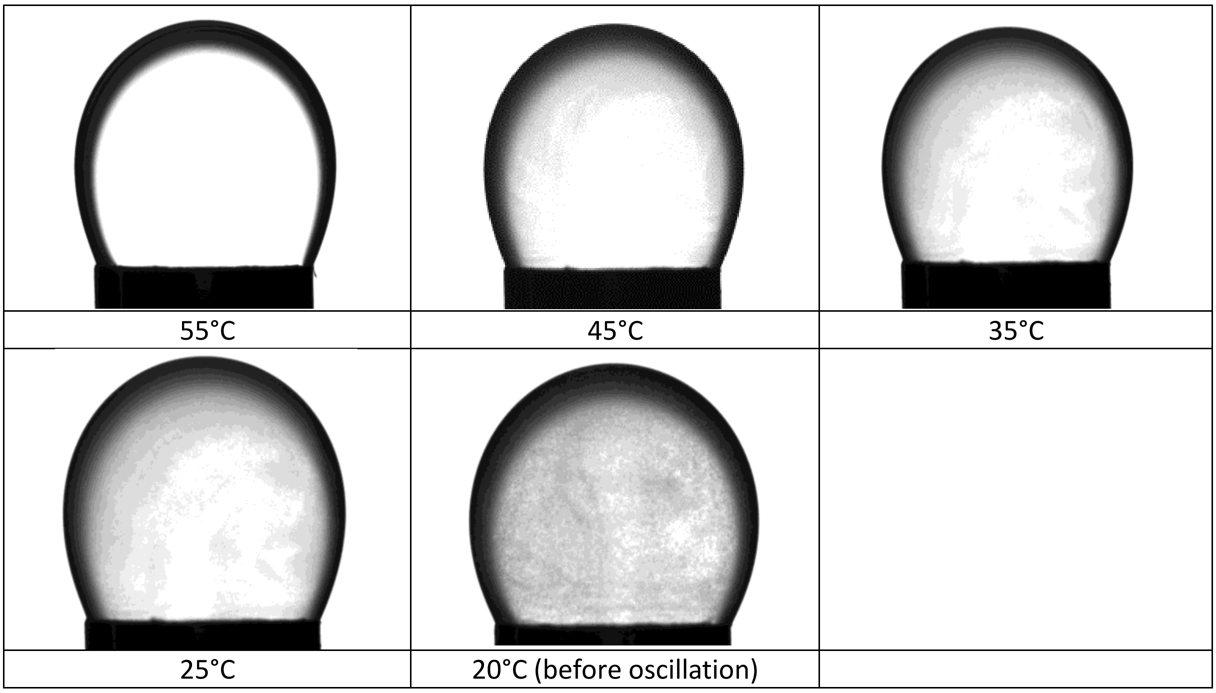

Changes of the oil droplet among Oscillation (Tween 60, BrijS20, Span 60)

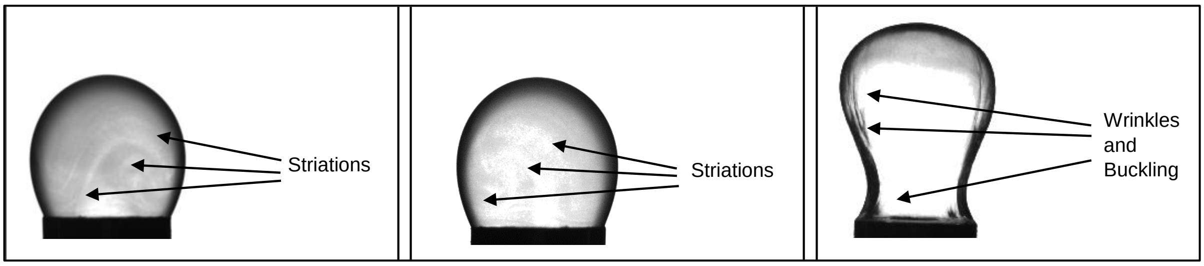

Stressing of the aged emulsifier interfacial layer on the MCT oil droplet by dilatation rheology stabilised by Tween 60 (left), BrijS20 (middle) and Span 60 (right) and using the droplet contour in emergent drop mode at T = 20°C.

<u>Monitoring of the development of Ed 'and Ed "of Span 60 during cooling</u>

The development of the Ed' and Ed" of the Span 60 stabilised oil-water interface was tracked by rising drop tensiometry (Tracker, Teclis Scientific) within a third experimental trial. At the beginning of the measurement (drop formation), the temperature was first set to 55 °C, and 30 minutes later, the temperature decreased steadily from 50 °C to 20 °C with a cooling rate of 0.5°C/min to simulate the melt emulsification process. The temperature was then held at 20 °C till the end of the measurement. During the whole measurement, a constant oscillation with an amplitude of 3% and a frequency of 0.01 Hz was put on the drop to gain information about the

development of Ed' during the cooling step, i.e. to catch the moment of phase transition. The calculation of Ed' was calculated at different temperature points, taking the changed density into account (recalculation option of the software, based on the saved pictures).

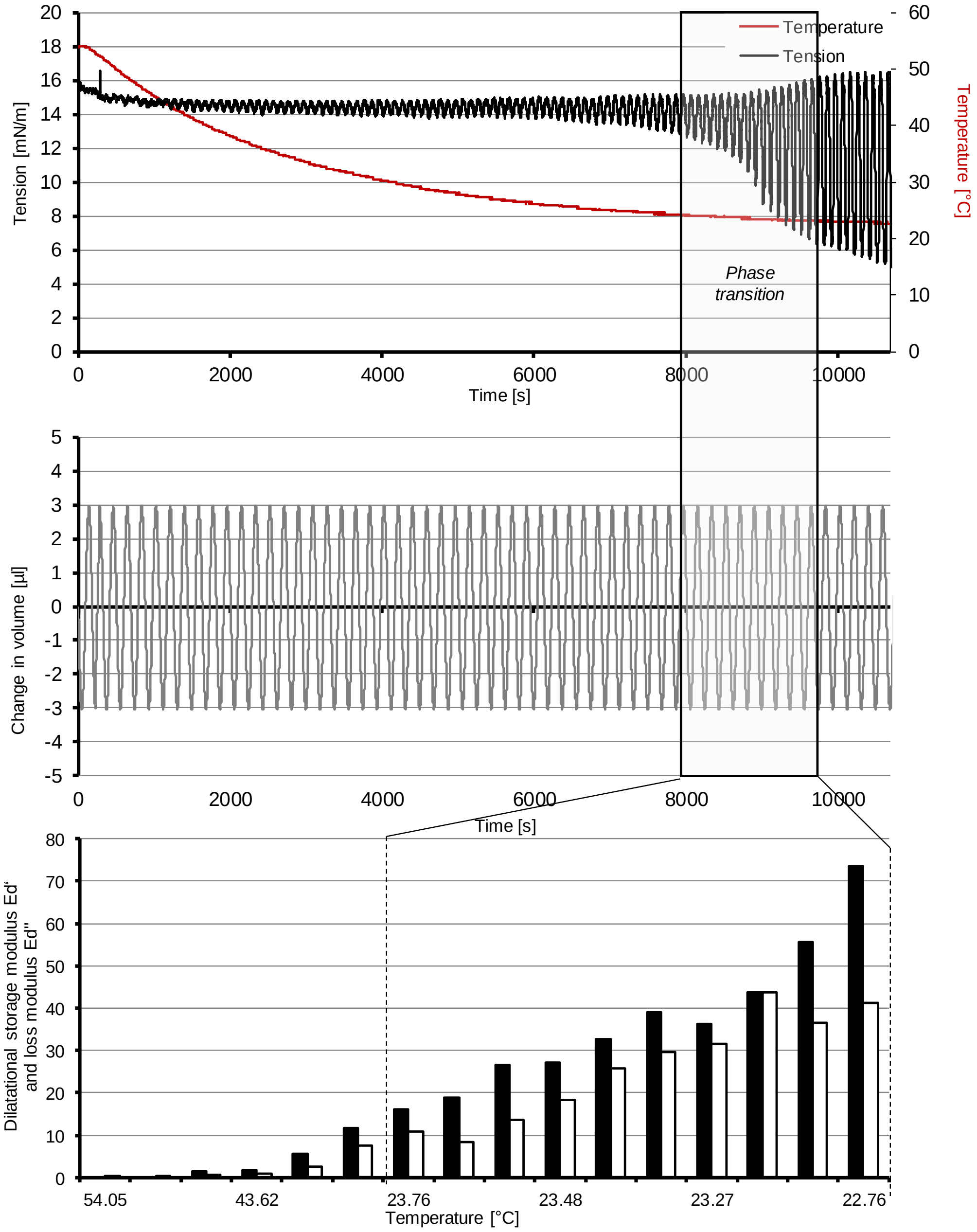

Monitoring of the development of Ed 'and Ed ''of Span 60 during cooling (T 55°C to 20°C). Top: Interfacial tension values as a function of temperature. Middle: Sinusoidal Oscillation (change if volume) during the cooling step. Bottom: Corresponding Storage and Loss modulus at the high temperature as well as during the phase transition region. Black bars: storage modulus. White bars: Loss modulus

Interfacial shear rheology

Frequency sweep of Span 60

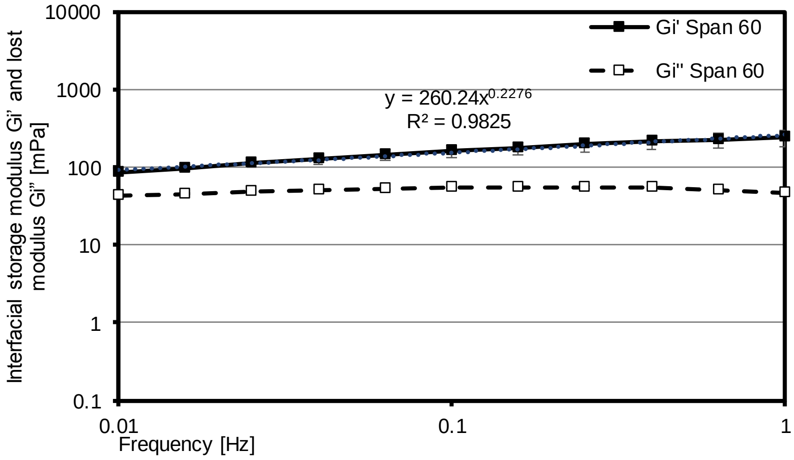

Storage modulus Gi'' and Gi'''' [mPa] of Span 60 over frequency, with a fixed amplitude of 0.1% using interfacial shear rheology at T = 20°C.

DSC measurements

Tween 20

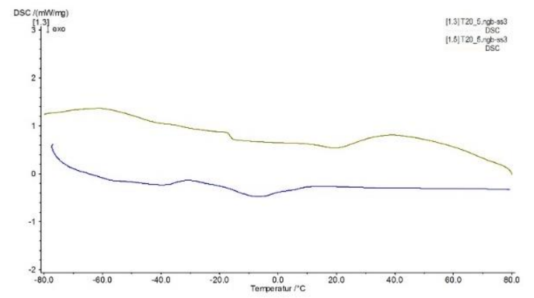

Span 20

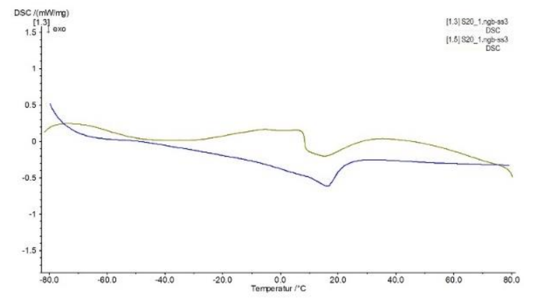

Tween 60

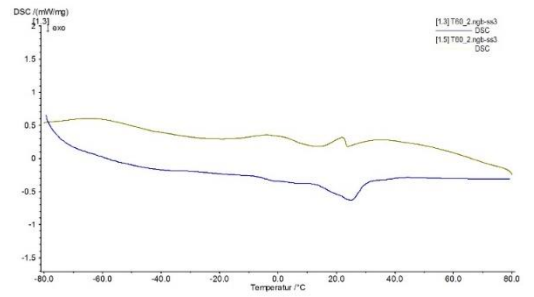

Span 60

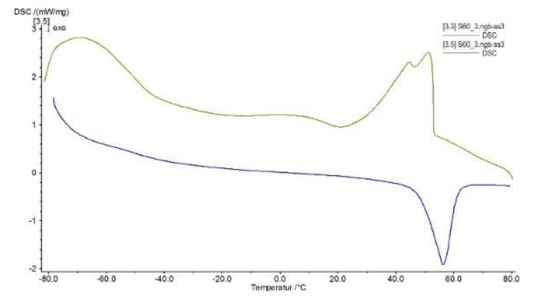

BrijS20

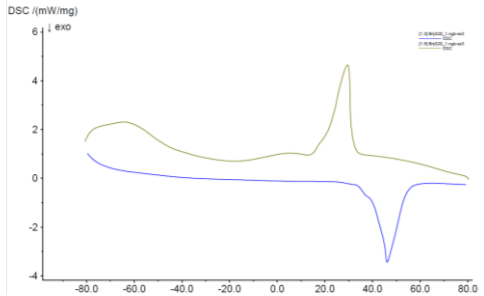

Tween 80

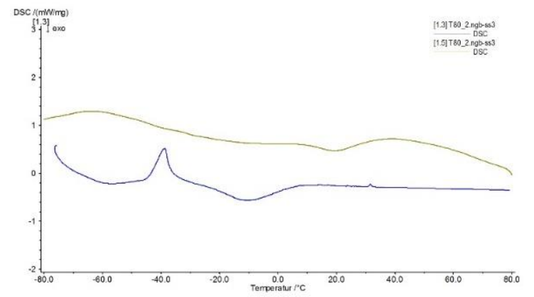

Span 80

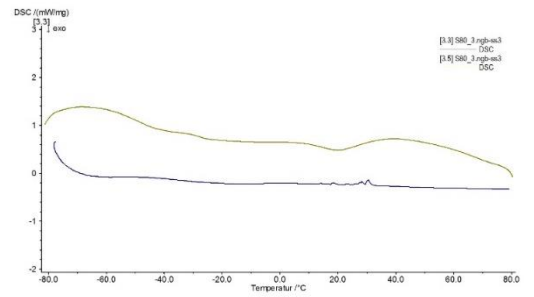

MCT oil

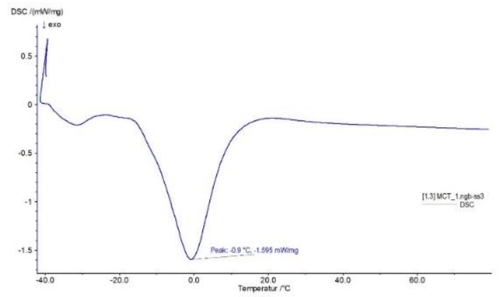

Melting and crystallisation curves of the surfactants. The surfactants were measured directly without dissolving them in a solvent first.
